# Examining Neurodiversity and Inclusion in Neuroscience Research Networks: A Case Study of the AIMS-2-TRIALS Autism Research Consortium

**DOI:** 10.12688/openreseurope.19394.1

**Published:** 2025-05-06

**Authors:** Teresa Del Bianco, Georgia Lockwood Estrin, Alexandra Lautarescu, Bethany F. Oakley, Eliza Eaton, Jerneja Terčon, Sarah Douglas, Jan Roderik Derk Plas, David Belton, Eva Loth, Mary Doherty, Emily J.H. Jones

**Affiliations:** 1 School of Social Sciences and Professions, London Metropolitan University, London, England, UK; 2 Centre for Brain and Cognitive Development, Birkbeck University of London, London, England, UK; 3 School of Psychology, University of East London, London, England, UK; 4 Department of Forensic and Neurodevelopmental Sciences, King's College London Institute of Psychiatry Psychology & Neuroscience, London, England, UK; 5 Communication Team, University of Cambridge Department of Psychology, Cambridge, England, UK; 6 AIMS-2-TRIALS A-Reps, University of Cambridge Department of Psychology, Cambridge, England, UK; 7 Department of Developmental Paediatrics and Early Intervention, Community Health Centre Domzale, Domzale, Slovenia; 8 Department of Neuroscience, Brighton and Sussex Medical School, Brighton, UK

**Keywords:** Autism, neurodiversity, neuroscience, research, representation

## Abstract

**Background:**

Due to the increased emphasis on co-produced and community led research, neurodiversity within research communities has sparked interest, particularly within the context of autism research. This study investigates the presence of neurodivergent researchers within a neuroscience research consortium, with a particular focus on autism prevalence.

**Methods:**

Using survey data collected from active contributors to the consortium, we examined the self-reported neurodivergent status of researchers, including formal diagnoses of autism, ongoing diagnostic processes, and self-identification as neurodivergent.

**Results:**

A proportion of the surveyed researchers reported formal diagnoses or self-identification as autistic (23%), that were significantly more frequent at career stages below and including postdoctoral roles (Chi-Square p-value = 0.01). Further, we identified an association between neurodivergence and a diagnosis of a mental health condition among researchers (Coef. = 1.93, p-value = 0.002), highlighting the importance of accommodating neurodiversity within research environments.

**Conclusions:**

This study underscores the need for inclusivity and support for neurodivergent researchers, particularly in the context of neuroscience that does or does not yet embed participatory research initiatives. By amplifying the voices of neurodivergent researchers, research communities can enhance the equity and impact of their outcomes and foster better public engagement by sharing experiences and understanding the needs of community members.

## Plain Summary

This study explores the growing interest in neurodiversity within research communities, particularly in autism research, as more projects focus on co-production and community-led approaches. It looks at how many researchers in a neuroscience consortium are neurodivergent, especially autistic.

Survey data from active researchers in the consortium showed that autism is fairly common among them, with 23% either formally diagnosed or self-identifying as autistic. This percentage was higher among researchers at career stages up to and including postdoctoral roles. The study also found that neurodivergent researchers reported lower mental well-being, highlighting the need for research environments to better support neurodiversity.

The findings emphasize the importance of creating inclusive and supportive spaces for neurodivergent researchers, particularly in participatory research. Amplifying their voices can make research outcomes more equitable, improve public engagement, and help the community better understand and address diverse needs.

## Introduction

Societal changes, such as the emergence of the Neurodiversity Paradigm ( Pellicano & den Houting, 2022), along with the advocacy efforts of individuals with neurodevelopmental conditions, are increasingly empowering neurodivergent populations ( Doyle, 2024) in neuroscience research. This shift is particularly impactful within the autistic community, where these changes are fostering greater inclusion and representation. Within this context, the autistic community has played a pivotal role in igniting and advancing discussions surrounding inclusion and decision-making in research, thereby shaping the research process from inception to dissemination ( Heraty *et al.*, 2023). However, amidst these critical discussions, the neurodivergent identities of researchers themselves have often been overlooked ( Abubakare, 2022; Botha, 2021). To contribute and enrich the ongoing debates surrounding inclusivity, representation, and decision-making within research communities, this work aims to assess the representation of neurodivergent researchers within one of the largest existing autism research consortia.

AIMS-2-TRIALS (Autism Innovative Medicine Studies-2-Trials) is the world's largest autism research grant, funding a multidisciplinary European consortium focused on autism research. This consortium primarily involves neuroscientists, health professionals, and psychologists. Its core goals are to advance the understanding of heterogeneity in individual profiles/needs, and developing innovative support measures for autistic people via large-scale studies and clinical trials that combine clinical assessments with neuroimaging and genetics.

Since its inception in 2018, the consortium has been an important driving force in progressing autism research, publishing 292 papers by 2023 (2.5 times higher than the world average; Innovative Health Initiative, 2022), highly cited and impactful results (for example, Begum Ali *et al.*, 2020; Bölte *et al.*, 2019; Lombardo *et al.*, 2019; Moessnang *et al.*, 2020; Pretzsch *et al.*, 2019), conducting clinical trials ( Del Bianco *et al.*, 2023), and delivering submissions to the European Medical Agency ( European Medicines Agency, 2020).

Operating across 14 countries, the consortium has enabled the harmonization and combination of data from smaller-scale, local studies within AIMS-2-TRIALS and multi-site data from various locations (e.g., Del Bianco *et al.*, 2024; Loth *et al.*, 2017). Additionally, it has facilitated multidisciplinary collaborations both within the consortium and with external partners (e.g., Del Bianco *et al.*, 2023), promoting shared practices and data, and generating joint publications. Besides researchers and project staff, the consortium includes a group of autistic representatives (A-Reps) – autistic adults and caregivers of autistic people – to advise on research priorities and goals, study design, ethics, and co-author publications from the consortium ( Heraty *et al.*, 2023).

Over the duration of this consortium (from its predecessor EU-AIMS funded in 2012, and AIMS-2-TRIALS since 2018) there have been substantial societal shifts in the perception and reception of autistic and other neurodivergent people. Both public and expert communities have questioned the legitimacy of neurodivergence research conducted exclusively by non-neurodivergent individuals, who lack the lived experiences of their research domain. This oversight may lead to the neglect of perspectives from neurodivergent populations ( Fletcher-Watson *et al.*, 2019; Levac *et al.*, 2019; Yusuf & Elsabbagh, 2015). Within this context, it has been suggested that biomedical methodological frameworks, including those within AIMS-2-TRIALS, adopt participatory models. These models aim to involve both neurodivergent and non-neurodivergent individuals at every stage of the research process. For instance, within AIMS-2-TRIALS, this involvement included the participation of A-Reps to the research process ( Heraty *et al.*, 2023). However, less attention has been paid to the existence of neurodivergent people within the existing research workforce. Individual researchers have shared their experience of neurodivergence with the public ( Abubakare, 2022; Botha, 2021), while certain principal investigators have begun prioritizing the recruitment of professionals who openly identify as autistic. Some scientific journals and conferences have even started asking for statements of neurodiversity from submitting authors and attendees (see ‘Community involvement statement’ in Jacques *et al.*, 2022). Nonetheless, disclosure is far from systemic in the research world, and the topic has become controversial, with researchers pointing out that those who are neurodivergent may not feel comfortable discussing their identities openly and/or within their work sphere ( Jacques *et al.*, 2022).

Here, we conducted a survey within the AIMS-2-TRIALS consortium with the goal of assessing the representation of neurodivergent researchers, whilst maintaining researchers’ privacy.

As such, this paper holds broader significance within the context of participatory research principles, particularly the ethos of ‘nothing about us without us’ ( Hoekstra *et al.*, 2018), highlighting the importance of including neurodivergent researchers in shaping research agendas. Furthermore, it will shed light on how the perspectives and experiences of neurodivergent researchers intersect with other intersectional identities, such as career distribution, and the relationship between mental health and their neurotype as compared to other socio-demographic variables. Specifically, career distribution will indirectly assess whether neurodiverse perspectives are represented throughout the research process, from those in early career roles who carry out the research to those in more senior positions who strategically plan and more directly shape research agendas. Secondly, investigating the relationship between mental health and neurotype in this sample will inform broader discussions about the strengths and challenges faced by neurodivergent researchers ( Dwyer *et al.*, 2021; Grant & Kara, 2021), and how these intersect with other risk factors, such as socio-economic status, for identifying potential reasons for disparities in career advancement and well-being.

## Methods

Our Methods, Results and Limitations sections and supplementary material available online (see Data Availability) follow best practices to ensure reproducibility by providing sufficient detail on materials and methods, in accordance with Kelley *et al.* (2003) and consensus-based reporting guidelines. Where applicable, we adhere to the EQUATOR Network guidelines ( https://www.equator-network.org/) to enhance transparency and methodological rigor.

### Participants

The participants to this study include researchers and/or student and staff members of the research consortium AIMS-2-TRIALS, including clinical, research and project personnel at various career levels, e.g., from professors to interns. Recruitment was carried out in January 2022 through the AIMS-2-TRIALS internal mailing list, with a four emails inviting people actively contributing to the design, execution, and/or analysis of AIMS-2-TRIALS studies to complete the survey.

### Tools

We created an in-house survey including multiple choice and open-ended questions that we distributed via the secure platform REDcap. Questions were collaboratively developed by four of the authors (TDB – who is a neurodivergent researcher –, GLE, EJHJ – who are researchers – and MD – who is a neurodivergent researcher and an A-rep) and revised by the AIMS-2-TRIALS Communication team, who manages and facilitates internal and external communication, public relations, media outreach, and dissemination of information to various stakeholders. The 47 questions included in the survey covered demographics, neurodiversity, mental health, and socio-economic status. Each question offered a "Prefer not to say" response option and could also be left unanswered. A detailed list of the questions and options for responses is available online (see section Data Availability). 

### Procedure

Ethical approval for this study was obtained from the Departmental Ethics Committee of the Department of Psychological Sciences at Birkbeck College, University of London. Members of AIMS-2-TRIALS received the invitation and link providing access to the survey via email from the AIMS-2-TRIALS Communication Team in January 2022. Comprehensive details regarding the study aims and procedures were provided in both the email and the study information sheet displayed after clicking the link. Participants could proceed to the consent at the bottom of the information screen. After confirming consent to participate, participants could proceed to the survey webpage and progress between questions.

### Measures and Statistical Analysis

As an overview, we report essential demographics such as means and standard deviation (i.e., for age) and percentages (e.g., for gender and career level) of the respondents. To increase the samples sizes at the level of individual categories and avoid low frequencies that can bias the results of the tests outlined below, we collapsed the 10 job titles available to respondents (see Table S1 for the complete list) into 3 career pathways (i.e., admin, clinician, and researcher). However, we kept the separation between early career researcher roles (ECRs, defined as research assistant, PhD student, research fellow, postdoctoral researcher) and senior researcher roles (defined as lecturer, reader, professor, director). While these researcher roles are not ordinally ordered and may not reflect the number of years in academia, the postdoctoral researcher status constitutes, in most countries, the career level prior to tenure, thus defining an earlier career stage.

To assess the distribution of autistic and non-autistic researchers across career pathways and roles, and whether the distribution is characterised by over/under-representation of neurodivergent researchers within specific roles, we performed a Chi-Square Test of association. A significant Chi-Square Test statistic, and the sign and magnitude of the difference between expected and observed frequencies within each subcategory (e.g., ECR and Autistic vs ECR and Neurotypical) are indicative of disparities in roles distribution. If the analysis included one or more expected frequencies below 5, we applied Yates' correction to the chi-square statistic to reduce the chance of obtaining a false positive (Type I error). We report the chi-square statistics, p-values, and the differences between observed and expected frequencies for significant pathway and role combinations to identify which pair of variables drives the association.

To assess the impact of being neurodivergent on having a diagnosis of a mental health condition, we used generalised multiple linear regression. This technique identifies which factors significantly contribute to the outcome, as well as quantifying the strengths of the association, and the contribution of individual factors. For the outcome variable, we used lifetime diagnoses of mental health conditions (binary, 0 / 1), and for the independent variables neurotype, gender, age, and proxies of socio-economic status that have been found to significantly weight on factors mapping onto the multifactorial nature of SES in a large sample of autistic and non-autistic people across various countries ( Del Bianco *et al.*, 2024; the proxies: living arrangement, number of bedrooms, number of household children, number of non-household adults financially supported, and subregion of residence; Southern Europe, Northern Europe, North America, Southern Africa, Eastern Africa). To increase the group-level sample size and avoid low frequencies, the variable neurotype combined responses to the questions “Are you autistic?” and “Do you have another developmental condition?”. These were grouped into two levels: neurodivergent (formal autism diagnosis, in the process of autism diagnosis, self-diagnosed, with another diagnosis of a neurodevelopmental condition) and neurotypical (answered no to both these questions). Examples were provided to participants to clarify the latter question (e.g., ADHD, dyslexia, dyspraxia). We report the coefficient estimates, standard errors, z-values, and p-values for significant predictors in the text, and for each independent variable in Table 1.

**Table 1.** Complete results of the generalised logistic regression, with formal mental health diagnosis as dependent variable.

	Estimate	SE	Z-Value	P-Value
Intercept	14.17	3956.18	0.003	0.99
Neurodevelopmental Conditions (Autism + Other)	1.93	0.63	-3.05	0.002*
Age	0.002	0.03	0.07	0.93
Gender (Woman)	1.46	0.75	1.93	0.05
Country of Residence (North America)	-31.90	4832.41	-0.006	0.99
Country of Residence (Northern Europe)	-16.66	3956.18	-0.004	0.99
Country of Residence (Southern Africa)	-17.32	3956.18	-0.004	0.99
Country of Residence (Southern Europe)	-17.05	3956.18	-0.004	0.99
Country of Residence (Western Europe)	-16.25	3956.18	-0.004	0.99
Living Arrangement (on my own)	1.32	1.19	1.102	0.27
Living Arrangement (with partner)	0.97	1.05	0.92	0.35
Living Arrangement (with parents)	35.24	4661.28	0.007	0.99
Living Arrangement (Other)	17.03	3956.18	0.004	0.99
Accommodation (Private Rental)	0.70	0.65	1.075	0.28
Accommodation (Social Housing)	-17.46	2464.99	-0.007	0.99
N of Bedrooms in Home	0.14	0.22	0.651	0.51
N of Children	0.13	0.39	0.33	0.73
N of Supported Adults	0.3111	0.51	0.59	0.54

## Results

The overall number of respondents was 124 (for a full breakdown of sample sizes, means and percentages for each question, see Tables S2-3 of the Supplementary Material available online.). Below, we report percentages of valid answers for each question; please note that percentages may not add up because the number of valid answers varies slightly between questions. We report the number of valid responses for each question in the Supplementary Material available online (see section Data Availability).

**Figure 1.**
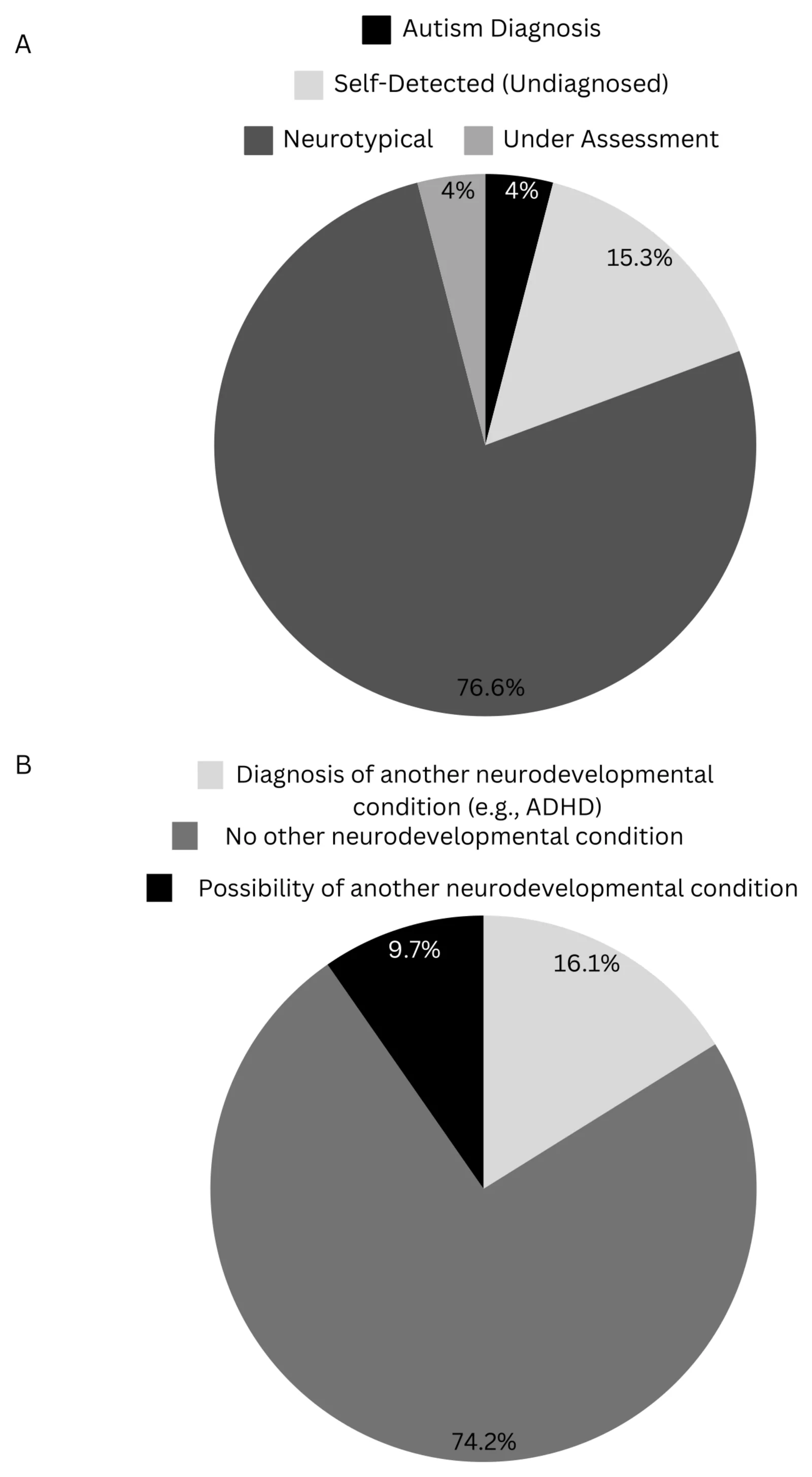
Percentages of respondents ( **A**) with autism formal diagnosis, undergoing assessment, self-detected, neurotypical. ( **B**) formal diagnosis of another neurodevelopmental condition, potential other neurodevelopmental condition, no other condition.

8.04% of respondents (N = 10) declared that they received a formal diagnosis of autism or are undergoing the diagnostic process; 15.32% (N = 19) responded that they think they are or might be autistic (self-detected), but never entered the diagnostic process, and 76.61% responded that they are not autistic, under assessment or think they are/might be autistic (see Figure 1 panel A). 16.13% (N = 20) responded that they have a formal diagnosis of another neurodevelopmental condition (e.g. ADHD, dyslexia, dyspraxia), while 9.68% (N = 12) responded that they are not sure (see Figure 1 panel 2). 30% (less than 5) of the formally diagnosed participants declared they had disclosed to their line manager. All self-detected respondents left this question blank.

The average age was 38.74 years (SD = 12.09, range 20 - 70). Most respondents were researchers (83.47%), followed by clinicians (10.4%), and administrative staff (3.47%). This figure aligned with the distribution of officially reported members of AIMS-2-TRIALS (researcher = 83%, clinician = 11%, admin = 7%; *AIMS-2-TRIALS - Autism Research For Europe*, 2018). The most common job title was postdoctoral researcher (N = 31, 25.62%), followed by research assistant (N = 19, 15.70%) and PhD student (N = 13, 10.74%), with ECRs being the most represented category overall (in line with officially reported figure of AIMS-2-TRIALS, ECRs = 49%; *AIMS-2-TRIALS - Autism Research For Europe*, 2018).

The most represented gender was female (N = 92, 74.19%), with a M:F ratio of ~5:16, in line with the official figure of gender representation of AIMS-2-TRIALS (M:F ratio ~7:13; *AIMS-2-TRIALS - Autism Research For Europe*, 2018). Most respondents were cis-gender (N = 121, 97.58%) with preferred pronouns she or he (N = 122, 98.38%, combined).

### Association between professional categories and neurodiversity (Chi-Square Tests)

**Figure 2.**
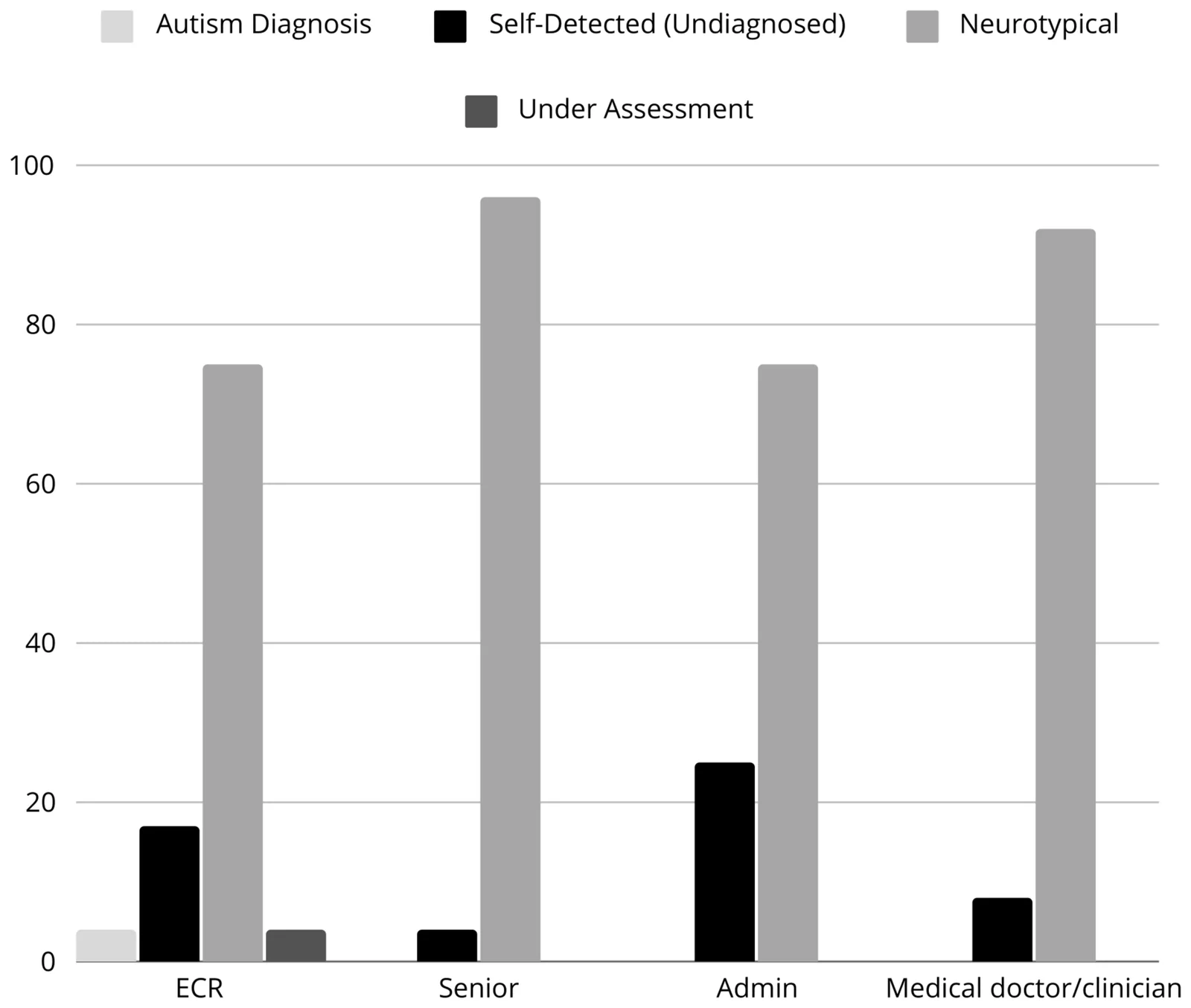
Graphical representation of professional category percentage distribution by neurotype.

There was a significant association between professional category and being autistic (Chi-Square with Yates correction for cells with low counts = 25.44, DF = 12, p-value = 0.01; see Figure 2 for a graphical representation of the distribution).

In terms of direction of these association, the difference between observed and expected frequencies was > 1 for respondents who self-diagnosed, meaning they were over-represented within the ECRs category (difference = 1.98), and conversely under-represented in senior researcher roles. On the other hand, neurotypical respondents were over-represented in the clinician (difference = 1.50) and senior researcher roles (difference = 4.00).

### Relationship with mental health diagnoses

**Figure 3.**
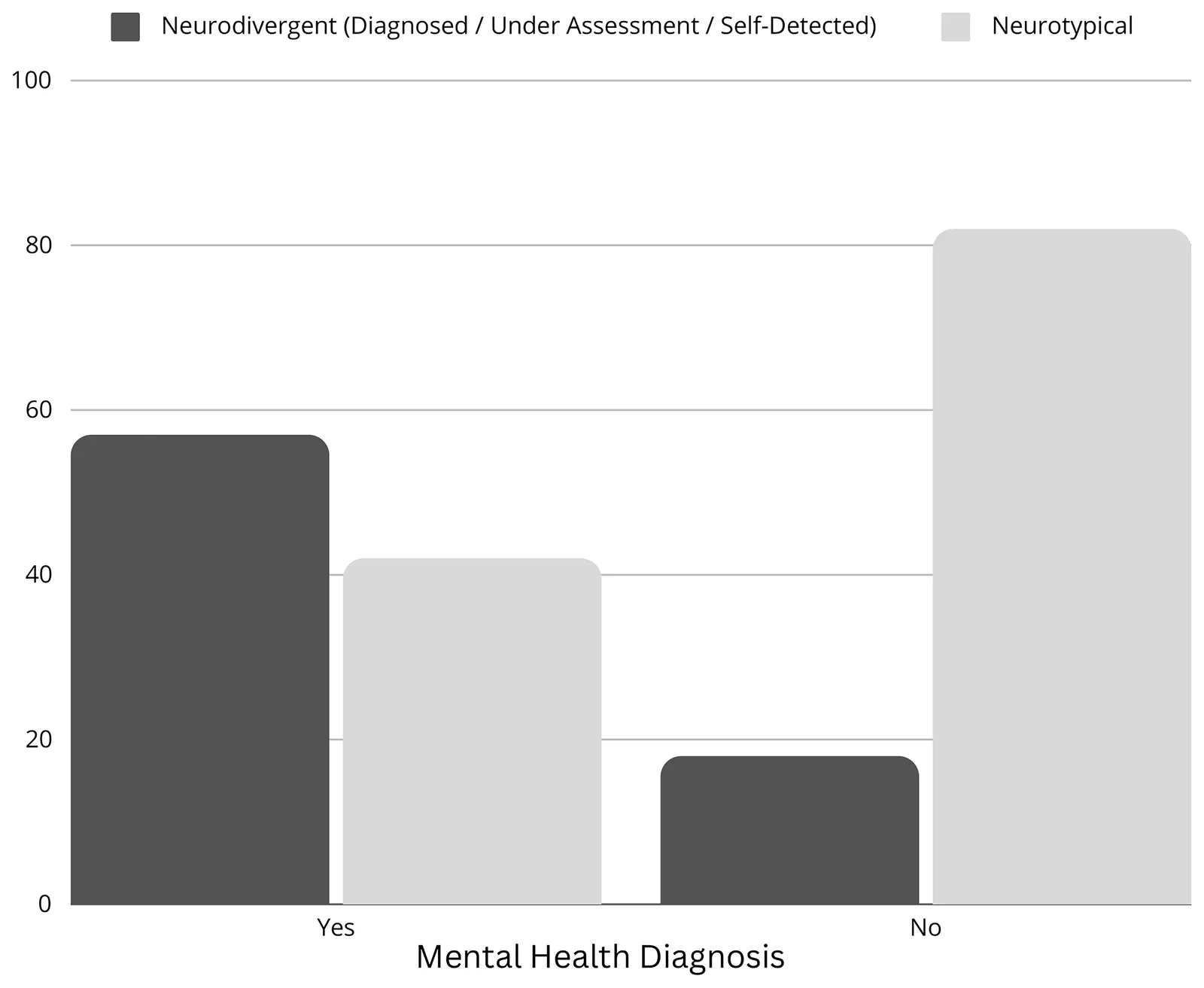
Graphical representation of the distribution of mental health diagnosis by neurotype.

The results of the generalised logistic regression ( Table 1) showed that the odds ratio of lifetime mental health diagnoses was significantly increased by being neurodivergent (diagnosed or self-identified, including autism and other neurodevelopmental conditions) compared to neurotypical (Coef. = 1.93, SE = 0.63, T-Value = 3.05, P-value = 0.002; see Figure 3 for a graphical representation). No other predictor (gender, age, socio-economic variables) turned out significant. The model showed good fit to the data, with a proportion of variance explained compared to baseline (based on between participants variability of lifetime mental health diagnosis, no predictors) of 0.86. The model did not violate the assumption of linearity and independence of predictors, and acceptable homoscedasticity of residuals (see Table S3 and Figure S1-2-3 of the Supplementary Material available online).

### Intersectionality

The percentage of females was higher among neurodivergent researchers (84.61%; M:F ~3:16) than neurotypical researchers (76.66%; M:F ~5:16). Although this difference was not significant (Chi-Square with Yates correction = 2.07, DF = 3, p-value = 0.55), gender was significantly associated with career level (Chi-Square = 16.97, DF = 4, p-value = 0.001; females over-represented in ECR). While non-significant, it is worth noting that the covariate gender increased the odds ratio of mental health diagnoses (Coef. = 1.46, p-value = 0.052), an observation that is in line with a large body of literature ( Li & Graham, 2017; Seedat *et al.*, 2009).

## Discussion

The primary goal of this study was to investigate the presence of neurodivergent researchers within the research consortium, focusing particularly on autism prevalence. Additionally, we aimed to explore how neurotype intersects with career distribution and mental health diagnoses among researchers. 4% of our participants reported a formal diagnosis of autism, 4% undergoing the diagnostic process, and 15% thinking they are autistic but have not pursued diagnosis. This prevalence was higher among earlier career roles. We also found an association between neurodivergence (including autism and other neurodevelopmental conditions, and formally diagnosed or self-detected) and higher odds of mental health diagnoses.

The 4% formal diagnosis rate in the consortium is higher than the worldwide (0.6%, 95% CI: 0.1–1; Salari *et al.*, 2022) and European prevalence (0.5%, 95% CI: 0.8–1.1; Salari *et al.*, 2022). Furthermore, it is well known that autism is underdiagnosed in women and individuals with lower support needs ( Kreiser & White, 2014; Smith *et al.*, 2019), who likely make up a significant portion of our sample. This demographic trend may explain why many contributors self-identify as autistic despite lacking a formal diagnosis.

These findings underscore the critical need to recognize and accommodate neurodiversity within the workforce of research consortia, especially those employing participatory approaches. In the pursuit of inclusivity, consortia may unintentionally overlook the perspectives of neurodivergent individuals already within their ranks while focusing on incorporating external contributors. It is essential to ensure that the voices of internal members, including those who are neurodivergent, are heard and valued alongside external perspectives. Neglecting to do so, albeit with the best intentions, may compromise the integrity of participatory research efforts. 

Additionally, the relatively low rate of formal disclosure among neurodivergent researchers in the consortium highlights the ongoing challenge of stigma and the need to foster an open and supportive environment. Efforts by AIMS-2-TRIALS to promote inclusivity have included webinars led by and for community members, researcher training on neuroaffirmative language, and research breaktime discussions with community members to foster collaboration. These initiatives likely contribute to reducing perceived stigma and creating a more open environment for neurodivergent researchers. However, further steps may be needed to ensure that researchers feel comfortable disclosing their diagnoses and fully participating in decision-making processes. These could include confidential disclosure mechanisms, proactive accommodations, peer support networks, and mandatory neurodiversity training.

### Association with career stage

The Chi-Square Test of association revealed that neurotype was significantly associated with career level; relative frequencies indicated that this association was driven by self-identified neurodivergent researchers that did not have an official diagnosis, who were under-represented in senior and clinical roles, compared to early career roles. This preliminary yet significant association reinforces the notion that career progression may be influenced by neurodivergent identity, with potential disparity stalling this group at earlier career stages. In turn, this finding may also be explained with generational differences reflecting evolving perceptions around neurodivergence, that may also play a role in determining real life outcomes, such as career progression. It remains to be clarified whether the weaker association between career level and formal diagnosis was due to the smaller sample size of the subcategory. This pattern may also be due to autistic adults being diagnosed at a later age, and/or increased neurodiversity among researchers that have been more recently employed by the consortium, and/or that a formal diagnosis helps researchers finding community, support and validation and boost their career. These possibilities may be disentangled in the future with a longitudinal, ad hoc study recruiting from several research consortia.

### Relationship with mental health diagnoses

The regression analysis identified neurodevelopmental conditions as significant predictors of increased likelihood of mental health diagnoses among the researchers. None of the other predictors (e.g., country of residence, living arrangement, number of children) were significant, showing that this relationship holds strong even when controlling for multiple risk factors. The co-occurrence of neurodivergence (not limited to autism, but also ADHD and dyslexia) and of mental health diagnoses such as anxiety and depression is a common and highly replicated finding ( Lai *et al.*, 2019; Lever & Geurts, 2016), that has been linked to multifactorial vulnerability that involves both genetics and environmental stressors ( Brito *et al.*, 2023). This result suggests mental health vulnerability amongst this group, and might point to need for additional support. From an employer’s perspective, recognizing the link between neurodivergence and mental health diagnoses might prompt them to provide more comprehensive support for both. For instance, if an employee discloses a neurodivergent condition, the employer could proactively offer mental health support, and vice versa.

### Intersectionality

***Dual Identity as Autistic and Autism Researcher***

One aspect to consider is the extent to which neurodivergent researchers can smoothly navigate their dual roles within participatory research. On one hand, they bring to the research process insights rooted in lived experiences ( Abubakare, 2022; Botha, 2021); on the other hand, they may face unique challenges related to disclosure, stigma, and accommodations ( Botha *et al.*, 2022; Love *et al.*, 2023). Their presence also raises the question about their role within participatory research (i.e., collaboration and shared decision-making between researchers and the communities they study). While neurodivergent researchers play a vital role in addressing power imbalances and epistemic injustice, it is essential to acknowledge that they hold a position of relative power within the academic structure. Genuine participatory efforts should therefore not only leverage their dual expertise but also actively involve community members outside of academia in shaping the research process. Drawing on established community-based participatory models from other fields (e.g., Anderson *et al.*, 2023; Gustafson & Brunger, 2014) could provide valuable guidance for fostering truly inclusive and collaborative research practices.

***Gender***

Even if outside of the scope of this study, the incidental finding of a significant association between career distribution and gender, overlapping with a higher representation of females among researchers, may indicate that the AIMS-2-TRIALS researchers experience overlapping forms of discrimination or privilege based on overlapping categories such as gender and neurotype. It is known that gender dynamics contribute to career progression and have an impact on mental health outcomes ( Bölte *et al.*, 2023). Future studies with larger samples should investigate how gender, neurotype, career level and other aspects of diversity influence researchers' experiences, including but not limited to mental wellbeing (e.g., access to resources and influence on decision-making). Extending the sample size would also allow to investigate this association within scientific communities with different gender balance (e.g., more males than females in some fields of neuroscience; Casad *et al.*, 2021).

### Limitations

One limitation of our survey study is that it was not obligatory to respond; however, the total number of respondents (N = 124) surpassed the count of the 2023 Consortium General Assembly attendees (N = 106). This could be indicative of potential attrition of researchers within the year following the circulation of the survey, but also account for the inclusion of researchers who were not present at the assembly due to personal circumstances and funding availability. Notwithstanding, the absolute number of autistic individuals in this sample is relatively small, thus cautioning any generalisation of our findings.

We report a slight difference (0.22) in the M:F ratio between our sample and the 2018 records. While we do not have a breakdown by career of the 2018 figure, the incidental finding that female researchers in our sample were over-represented in ECR roles might explain this slight deviation.

The distribution of the survey considered the possibility of respondent bias. Specifically, the respondents were informed that their responses would be kept confidential, and that all responses were to remain anonymous. All questions had the option of leaving them blank, so not forcing researchers to respond when they felt a piece of information could identify them (for example, their country of residence). These measures should have minimised the possibility that researchers who worry about disclosing their identity choose not to respond. A limitation we could not avoid was the circulation via the internal mailing list; however, while this might have excluded researchers who did not access their email during that time frame, it allowed us to make sure that researchers external to the consortium did not complete the survey, as their exclusion would not have been possible due to complete anonymisation.

### Representativeness

Overall, while there are potential limitations in the representativeness of the entire AIMS-2-TRIALS network, our results offer preliminary yet valuable insights into the characteristics of early career researchers who form the majority of this consortium and were recent active contributors. Insights from this group has the potential to inform which populations will influence the future trajectory of initiatives stemming from this consortium, as early career researchers trained under AIMS-2-TRIALS transition to leadership roles and move between institutions and countries. These findings are likely to apply more broadly to the next generation of neuroscience researchers, particularly those working within neurodevelopmental disorders. It is important to note that rates of neurodivergence may be higher in autism research compared to other areas of neuroscience, due to the *me-search* effect ( Devendorf *et al.*, 2023), highlighting the need to consider these factors across diverse research domains. These preliminary findings also suggest that those who are currently in leadership positions and communities could benefit from amplifying the voices of ECRs who are more likely to be neurodivergent and thus could influence decision making and goals setting from the perspective of lived experience.

While our survey was mostly quantitative, we did collect responses to two open questions regarding research goals: 'What do you perceive to be the goal of your research within AIMS-2-TRIALS?' and 'What do you think is the single most important goal for future autism research?' While the short questions and answers were not designed for in depth qualitative analysis, we report sampled responses in Table 2, which provide a glimpse into the perspectives of AIMS-2-TRIALS neurodivergent researchers on their research goals and the future of autism research. These comments, while constructive, reflect a desire for change and could indicate issues around researchers' sense of satisfaction and belonging. If researchers feel that their research goals are not aligned with what they believe they should be, they may start to feel out of place or less committed to their work. Addressing these insights should be a focal point for future research to better understand and support the well-being and inclusion of neurodivergent researchers. Furthermore, our regression analysis investigating mental health, which included various covariates such as living arrangements, number of bedrooms, number of household children, and number of non-household adults financially supported, provides additional context for understanding the well-being of neurodivergent researchers within the consortium. Mental health outcomes directly reflect well-being, making them a crucial focus. The open-ended questions offer additional clues about how meaningful researchers find their work and highlight areas where improvements could enhance their overall sense of satisfaction and inclusion.

**Table 2.** Selected responses to the two open ended survey questions, provided by respondents who had a formal diagnosis of autism, were in the process of obtaining one, or thought that they are or might be autistic. Note this is a subset of responses from a larger set that shared similarities, selected by the first author as they encapsulated the overarching concept being discussed by all provided responses.

*“What do you perceive to be the goal of your research within AIMS-2-TRIALS?”*
Understanding the autistic experience of life, reality, its behavioural scaffolding and neural underpinning
To contribute to diversity of opinion within a biomedical research context.
Help to improve the wellbeing of autistic people through understanding neurobiological mechanisms
Improve autistic lives. Decolonialise science.
More clarity on how better inclusion can be reached so all people can participate in society in their own way with ample opportunities. Better treatments for those who needs it. More understanding for all subentities.
Make more cohesive cooperation between autistic community and autism researchers.
Also move towards more inclusion of autistic individuals in design of studies, outcomes and so on. There is still a huge gap.
*“What do you think is the single most important goal for future autism research?”*
To bring awareness to Autism and help autistic people.
Overcoming the pathologisation of autism
Achieving equitable recognition of research priorities across all stakeholder groups, and corresponding parity of funding.
To include autistic people in the research process itself. Autism research requires genuine co-production rather than tokenistic inclusion in the research. Much more research along the lines of autistic peoples (and their allies) research priorities. However I do think that there is also a need for basic science autism research, but it is critical to find a better balance between these two (which at the moment definitely doesn't exist).
Help to improve the wellbeing of autistic people through understanding neurobiological mechanisms

## Conclusions

Beyond validating the existence of neurodivergent researchers within this consortium without invading their privacy, this work provides preliminary evidence about the career distribution of autistic researchers, an area worth investigating. Given the differential distribution between career levels, studying career progression with longitudinal approaches could offer insights into trajectories and experiences over time, to find out the factors influencing long term success and challenges. Furthermore, it could offer insights into the ongoing evolution of research communities, who have increasingly begun to shift towards involving neurodivergent people and recognising the existing involvement of neurodivergent people in the making of science. Considering cultural variations and other intersecting factors, such as ethnicity and gender, the comparison with others research consortia, e.g., in global settings, could help to fully understand the extent of this cultural shift. To investigate the causes of mental health issues and potentially delayed career progression, a follow up survey and future quantitative studies should be integrated with interviews and focus groups, that could provide vital information on the impact of being a researcher and neurodivergent on people’s quality of life. Composing this information into a clear picture of who is conducting research in the autism field could ultimately contribute to rebuilding the bridge between researched and researchers.

## Ethics and consent

Ethical approval for this study was obtained from the Departmental Ethics Committee of the Department of Psychological Sciences at Birkbeck College, University of London on 26/07/2021 (reference number: 2021091).

Members of AIMS-2-TRIALS received the invitation and link providing access to the survey via email from the AIMS-2-TRIALS Communication Team in January 2022. Comprehensive details regarding the study aims and procedures were provided in both the email and the study information sheet displayed after clicking the link.

Participants could proceed to the consent at the bottom of the information screen.

Participants provided written consent by confirming that:

6. They understood the details of the study as explained to them and willingly consented to take part.
7. They were aware that they could ask further questions to the study investigator via email at any time.
8. They understood that their participation would remain anonymous, and all information provided would be used solely for the purposes of the study.
9. They could decline to answer specific questions and withdraw their data at any time before submitting the questionnaire, after which the anonymized data could no longer be identified.
10. All information provided would be kept confidential, and no personally identifiable data (e.g., name, date of birth) would be collected.
11. Anonymous responses would be stored securely and accessed only by authorized researchers.
12. The results of the study would be written up for a project/thesis and presented at conferences and in academic journals. Any individual data presented would be completely anonymous, with no means of identifying participants.
13. They were 18 years or older.

After confirming consent, participants could proceed to the survey webpage and progress between questions.

## Abbreviations

AIMS-2-TRIALS: Autism Innovative Medicine Studies-2-Trials

A2T (in figures): AIMS-2-TRIALS

A-Reps: Autistic Representatives

ECRs: Early Career Researchers

Coef.: coefficient

SE: standard error

P-value: probability value

SD: standard deviation

## Data Availability

OSF: **Examining Neurodiversity and Inclusion in Neuroscience Research Networks: A Case Study of the AIMS-2-TRIALS Autism Research Consortium: Materials and Analysis**. osf.io/7pbvr ( OSF, 2024). The project contains the following underlying data: Analysis Script Ethics Materials Supplementary Material Data are available under the terms of the Creative Commons Attribution 4.0 International license (CC-BY 4.0). All the materials (including the information sheet, consent form and survey questions), analysis code and extended data have been made available on a public access repository at the following link: osf.io/7pbvr. We adopt a curated data-sharing approach, meaning that direct access to the data is limited to the study investigators. Other researchers interested in using this data must apply for access by submitting a brief research scope and a motivation letter, detailing the intended purpose of the data, via email to the corresponding author. The co-authors of this paper—including autism community members—will collectively evaluate requests and determine acceptable purposes on behalf of the study cohort. **This approach aligns with the specific security and privacy requirements outlined in the participants' consent for this study (available along with all study materials at the link above), as approved by the Departmental Ethics Committee of the Department of Psychological Sciences at Birkbeck College, University of London. Additionally, it is supported by evidence indicating that autistic individuals and their families prefer curated over open data-sharing (see Ashworth *et al.*, 2021; Begum Ali *et al.*, 2024; Hobson *et al.*, 2023).**
