# Neurodivergent Researchers in Autism Research: A Survey within the AIMS-2-TRIALS Consortium

**DOI:** 10.12688/openreseurope.19394.2

**Published:** 2026-07-09

**Authors:** Teresa Del Bianco, Georgia Lockwood Estrin, Alexandra Lautarescu, Bethany F. Oakley, Eliza Eaton, Jerneja Terčon, Sarah Douglas, Jan Roderik Derk Plas, David Belton, Eva Loth, Mary Doherty, Emily J.H. Jones

**Affiliations:** 1 School of Social Sciences and Professions, London Metropolitan University, London, England, UK; 2 Centre for Brain and Cognitive Development, Birkbeck University of London, London, England, UK; 3 School of Psychology, University of East London, London, England, UK; 4 Department of Forensic and Neurodevelopmental Sciences, King's College London Institute of Psychiatry Psychology & Neuroscience, London, England, UK; 5 Communication Team, University of Cambridge Department of Psychology, Cambridge, England, UK; 6 AIMS-2-TRIALS A-Reps, University of Cambridge Department of Psychology, Cambridge, England, UK; 7 Department of Developmental Paediatrics and Early Intervention, Community Health Centre Domzale, Domzale, Slovenia; 8 Department of Neuroscience, Brighton and Sussex Medical School, Brighton, UK

**Keywords:** Autism, neurodiversity, neuroscience, research, representation

## Abstract

**Background:**

Growing emphasis on participatory and community-informed research has increased interest in the representation of neurodivergent researchers within the research workforce. This exploratory study investigated the presence of neurodivergent researchers within a large European autism research consortium and examined associations with selected demographic, occupational, and mental health characteristics.

**Methods:**

An anonymous survey was distributed to active contributors within the consortium. Participants reported autism diagnosis status, self-identification, other neurodevelopmental conditions, demographic characteristics, career stage, and lifetime mental health diagnoses.

**Results:**

33% percent of participants met the study definition of neurodivergence, including researchers with a formal autism diagnosis, those undergoing diagnostic assessment, self-identified autistic researchers, and researchers reporting other neurodevelopmental conditions. Neurodivergent researchers were represented across all career stages within the consortium. Neurodivergence was associated with increased odds of reporting a lifetime mental health diagnosis after adjustment for age and gender. Bayesian sensitivity analyses produced consistent findings.

**Conclusions:**

These findings provide an initial characterisation of neurodivergent researchers within a large autism research consortium and underscore the importance of considering neurodivergent researchers as part of broader discussions on inclusion in autism research. Further research across multiple research settings is needed to better understand workforce experiences, disclosure, and support needs.

## Plain Summary

This study explores the growing interest in neurodiversity within research communities, particularly in autism research, as more projects focus on co-production and community-led approaches. It looks at how many researchers in a neuroscience consortium are neurodivergent, especially autistic.

Survey data from active researchers in the consortium showed that autism is fairly common among them, with 23% either formally diagnosed or self-identifying as autistic. This percentage was higher among researchers at career stages up to and including postdoctoral roles. The study also found that neurodivergent researchers reported lower mental well-being, highlighting the need for research environments to better support neurodiversity.

The findings emphasize the importance of creating inclusive and supportive spaces for neurodivergent researchers, particularly in participatory research. Amplifying their voices can make research outcomes more equitable, improve public engagement, and help the community better understand and address diverse needs.

**Abbreviations**
AIMS-2-TRIALS: Autism Innovative Medicine Studies-2-Trials
A-Reps: Autistic Representatives
ECRs: Early Career Researchers
Coef: Coefficient
SE: Standard error
P-value: Probability value
SD: Standard deviation

## Introduction

The emergence of the Neurodiversity Paradigm ( Pellicano & den Houting, 2022), which conceptualises neurological differences as natural forms of human variation rather than deficits, along with the advocacy of individuals with neurodevelopmental conditions, are increasingly empowering neurodivergent populations ( Doyle, 2024). Within this framework, *neurodiversity* refers to the diversity of human neurocognitive functioning across populations, while *neurodivergent* describes individuals whose neurological development or functioning differs from societal norms, including autistic people and those with other neurodevelopmental conditions ( Doyle, 2024). Within the context of neuroscience, the autistic community is playing a pivotal role in advancing discussions surrounding inclusion and decision-making in research, and shaping the research process from inception to dissemination ( Heraty *et al.*, 2023). Amidst these discussions, neurodivergent researchers have started to join the discussion providing insights into their role and challenges ( Abubakare, 2022; Botha, 2021). Within this context, this exploratory work aims to assess the representation of neurodivergent researchers as a group within one of the largest existing autism research consortia.

AIMS-2-TRIALS (Autism Innovative Medicine Studies-2-Trials) a large European autism research consortium that brings together researchers neuroscience, psychology, psychiatry, genetics clinical research, and industry and community partners across 14 countries ( AIMS-2-TRIALS, 2018). Established in 2018, the consortium aims to improve understanding of the heterogeneity of autism and to support the development of personalised approaches to intervention through large scale studies and clinical trials ( Oakley *et al.*, 2022). Beyond scientific outputs and in line with participatory research principles, the consortium has sought to incorporate autistic perspectives through the involvement of a group of autistic public representatives (A-Reps), who contribute to research prioritization, study design, ethics, dissemination, and publications ( Heraty *et al.*, 2023).

Over the duration of this consortium (from its predecessor EU-AIMS funded in 2012 and AIMS-2-TRIALS since 2018) there have been substantial societal shifts in the perception and reception of autistic and other neurodivergent people. Historically, autism research has been conducted by researchers, with autistic people having limited involvement in determining research priorities, study design and interpretation (CIT). Both public and scientific communities have questioned the legitimacy of neurodivergence research conducted exclusively by neurotypical individuals, who lack the lived experiences of their research domain ( Fletcher-Watson *et al.*, 2019; Levac *et al.*, 2019; Yusuf & Elsabbagh, 2015). The Neurodiversity Paradigm has further contributed to this shift by emphasising neurodivergent lived experience as a valuable source of expertise, rather than viewing autism solely through a deficit-based lens (CIT). Within this context, it has been suggested that biomedical methodological frameworks, including those within AIMS-2-TRIALS, adopt participatory models. These models aim to involve both neurodivergent and neurotypical individuals at every stage of the research process. For instance, within AIMS-2-TRIALS, this involvement included the participation of A-Reps to the research process ( Heraty *et al.*, 2023). However, less attention has been paid to the existence of neurodivergent people within the existing research workforce. Individual researchers have shared their experience of neurodivergence with the public ( Abubakare, 2022; Botha, 2021), and there is growing discussion within some research communities about actively supporting neurodivergent researchers (e.g., see for example the Neurodiversity and Participatory Research Advisory Group of the Respect4Neurodevelopment research community). Some scientific journals and conferences have even started asking for statements of neurodiversity from submitting authors and attendees (see ‘Community involvement statement’ in Jacques *et al.*, 2022). Nonetheless, disclosure is far from systemic in the research world, and the topic has become controversial, with researchers pointing out that those who are neurodivergent may not feel comfortable discussing their identities openly and/or within their work sphere ( Jacques *et al.*, 2022).

Here, we conducted a survey within the AIMS-2-TRIALS consortium to estimate the representation of neurodivergent researchers whilst maintaining participants’ privacy. Given the increasing emphasis on participatory approaches and the inclusion of neurodivergent perspectives in research ( Hoekstra *et al.*, 2018), understanding the composition of a research workforce represents and important, yet relatively underexplored, area of inquiry. The study aimed to describe prevalence and distribution of neurodivergence researchers within the consortium, and explore how neurotype relates to selected demographic and occupational characteristics, such as career stage, and self-reported mental health. As an exploratory investigation, the study was not designed to test specific hypotheses, but rather to provide an initial characterisation within this consortium and inform future discussions regarding diversity, inclusion and researcher well-being in autism research settings.

## Methods

Our Methods, Results and Limitations sections and supplementary material are available online (see Data Availability) and follow best practices to ensure reproducibility by providing sufficient detail on materials and methods, in accordance with Kelley *et al.* (2003) and consensus-based reporting guidelines. Where applicable, we adhere to the EQUATOR Network guidelines ( https://www.equator-network.org/) to enhance transparency. This study was exploratory in nature and was not pre-registered. Analyses were designed to provide an initial description of the prevalence and distribution of neurodivergent researchers within the consortium and to explore associations between neurotype and selected and occupational characteristics.

### Participants

The participants to this study include researchers and/or student and staff members of the research consortium AIMS-2-TRIALS, including clinical, research and project personnel at various career levels, e.g., from professors to interns. Recruitment commenced in January 2022 through the AIMS-2-TRIALS internal mailing list. During the recruitment period, and initial invitation and subsequent reminder emails were distributed inviting individuals actively contributing to the design, execution, and/or analysis of AIMS-2-TRIALS studies to complete the survey.

### Tools

We created an in-house survey including multiple choice and open-ended questions that we distributed via the secure platform REDcap. Questions were collaboratively developed by four of the authors (TDB – who is a neurodivergent researcher –, GLE, EJHJ – who are researchers – and MD – who is a neurodivergent researcher and an A-rep) and revised by the AIMS-2-TRIALS Communication team, who manages and facilitates internal and external communication, public relations, media outreach, and dissemination of information to various stakeholders. The 47 questions included in the survey covered demographics, neurodiversity, mental health, and socio-economic status. Each question offered a “Prefer not to say” response option and could also be left unanswered. A detailed list of the questions and options for responses is available online (see section Data Availability).

### Procedure

Ethical approval for this study was obtained from the Departmental Ethics Committee of the Department of Psychological Sciences at Birkbeck College, University of London. Members of AIMS-2-TRIALS received the invitation and link providing access to the survey via email from the AIMS-2-TRIALS Communication Team in January 2022. Comprehensive details regarding the study aims and procedures were provided in both the email and the study information sheet displayed after clicking the link. Participants could proceed to the consent at the bottom of the information screen. After confirming consent to participate, participants could proceed to the survey webpage and progress between questions.

### Measures and statistical analysis

The following analyses were exploratory in nature and were not pre-registered. Analyses were designed to provide an initial description of the prevalence and distribution of neurodivergent researchers within the consortium and to explore associations between neurotype and selected demographic, occupational, and mental health characteristics. Accordingly, all analyses should be interpreted as exploratory rather than confirmatory.

Unless otherwise specified, unanswered questions were treated as missing values (NA) and were not imputed. Analyses were conducted using available-case data, such that participants with missing data for a given variable were excluded only from analyses involving that variable. The only exception concerned three free-text occupational responses, which were reclassified into existing career categories based on the reported primary role (see below).

We calculated essential demographics such as means and standard deviation (i.e., for age) and percentages (e.g., for gender and career level) of the respondents. To increase the sample sizes at the level of individual categories and sparse cells, we collapsed the 10 job titles available to respondents (see Table S1 for the complete list) into 3 career pathways (i.e., admin, practitioner, and researcher). Researcher referred to individuals primarily engaged in the design, conduct, analysis, or dissemination of research. Practitioner referred to individuals primarily engaged in clinical or professional practice (e.g., psychologists, psychiatrists, speech and language therapists), whether or not they also contributed to research activities. Admin referred to individuals primarily engaged in administrative, operational, or project management roles supporting the research consortium. We kept the separation between early career researcher roles (ECRs, defined as research assistant, PhD student, research fellow, postdoctoral researcher) and mid-level and senior researcher roles (defined as lecturer, reader, professor, director). While these researcher roles are not ordinally ordered and may not reflect the number of years in academia, the postdoctoral researcher status constitutes, in most countries, the career level prior to tenure, thus defining an earlier career stage. Free-text responses entered under “Other” were reviewed and reclassified where the reported occupation could be assigned to an existing category. Responses that did not describe an occupation (e.g., reporting a qualification instead of a professional role) were treated as missing for this variable.

For the statistical analyses, responses to the questions “Are you autistic?” and “Do you have another developmental condition?” were combined into a single neurotype variable. Participants were classified as neurodivergent if they reported a formal autism diagnosis, being in the process of obtaining an autism diagnosis, self-identification as autistic, or responded “yes” or “maybe/I am not sure” to the question regarding another developmental condition. Participants who answered “no” to both questions were classified as neurotypical. Examples of other developmental conditions (e.g., ADHD, dyslexia, and dyspraxia) were provided within the survey to clarify the latter question.

To assess the distribution of autistic and neurotypical researchers across career pathways and roles, we performed a Chi-Square Test of association in R (Posits Studio, 2025). To aid interpretation of the analysis, sensitivity power analyses were conducted in G*Power ( Faul *et al.*, 2009). With N = 124, df = 12, α = .05 and 80% power, the minimum detectable effect size was *w* = 0.37. Differences between observed and expected frequencies (i.e., standardized residuals) were examined to determine which career pathways and roles contributed most to the overall association. We report the chi-square statistics, p-values, and standardised residuals for career pathway and neurotype combinations.

To assess the impact of being neurodivergent on having a diagnosis of a mental health condition, we used a generalized linear model with a binomial error distribution and logit link function in R. The outcome variable was lifetime diagnoses of mental health conditions (binary, 0/1); the predictor variables were neurotype, gender, and age. We report the coefficient estimates, standard errors, z-values, and p-values for significant predictors in the text, and for each independent variable. As a sensitivity analysis, we repeated the model using a Bayesian logistic regression with the same predictors using the brms package in R ( Bürkner, 2017). The model assumed a Bernoulli likelihood with a logit link and was fitted using four Markov chain Monte Carlo chains with 4,000 iterations each (2,000 warm-up iterations), using the default weakly informative priors. Convergence was assessed using the potential scale reduction factor (R ^ ), effective sample sizes (Bulk and Tail ESS), and posterior predictive checks.

## Results

The overall number of respondents was 124 (see Table 1; for a full breakdown of sample sizes, means and percentages for each question, see Tables S2–3 of the Supplementary Material available online). Below, we report percentages of valid answers for each question; please note that percentages may not add up because the number of valid answers varies slightly between questions. We report the number of valid responses for each question in the Supplementary Material available online (see section Data Availability).

The average age was 38.74 years (SD = 12.09, range 20–70). Most respondents were researchers (83.47%), followed by clinicians (10.4%), and administrative staff (3.47%). This figure aligned with the distribution of officially reported members of AIMS-2-TRIALS (researcher = 83%, clinician = 11%, admin = 7%; *AIMS-2-TRIALS - Autism Research For Europe*, 2018). The most common job title was postdoctoral researcher (N = 31, 25.62%), followed by research assistant (N = 19, 15.70%) and PhD student (N = 13, 10.74%), with ECRs being the most represented category overall (in line with officially reported figure of AIMS-2-TRIALS, ECRs = 49%; *AIMS-2-TRIALS - Autism Research For Europe*, 2018).

The most represented gender was female (N = 92, 74.19%), with a M:F ratio of ~5:16, in line with the official figure of gender representation of AIMS-2-TRIALS (M:F ratio ~ 7:13; *AIMS-2-TRIALS - Autism Research For Europe*, 2018). Most respondents were cis-gender (N = 121, 97.58%) with preferred pronouns she or he (N = 122, 98.38%, combined).

Among respondents, 5 (4%) reported a formal autism diagnosis, 5 (4%) reported currently undergoing diagnostic assessment, and 19 (15%) reported that they believed they were autistic but had not pursued a formal diagnosis. Regarding other developmental conditions, 20 (16%) reported having another developmental condition and a further 12 (10%) responded “maybe/I am not sure.” For the inferential analyses, these responses were combined into a single neurotype variable, yielding 42 neurodivergent (33%) and 82 neurotypical (66%) participants ( Figure 1). Because participants could report both autism and another developmental condition, the autism and other developmental condition categories were not mutually exclusive. Consequently, the neurodivergent category represents the union of these responses rather than their sum.

**Table 1.** Complete results of the generalised logistic regression, with formal mental health diagnosis as dependent variable.

Characteristic	n (%) or Mean (SD)
Age	38.74 (12.09)
Female	92 (74.19%)
Male	29 (23.39%)
Non-binary/self-describe	2 (1.61%)
Formal autism diagnosis	5 (4.03%)
Undergoing autism assessment	5 (4.03%)
Self-identified autistic	19 (15.32%)
Other developmental condition	20 (16.13%)
Possible developmental condition	12 (9.68%)
Neurodivergent (analysis variable)	42 (34%)
Lifetime mental health diagnosis	40 (34.78%)

**Figure 1.**
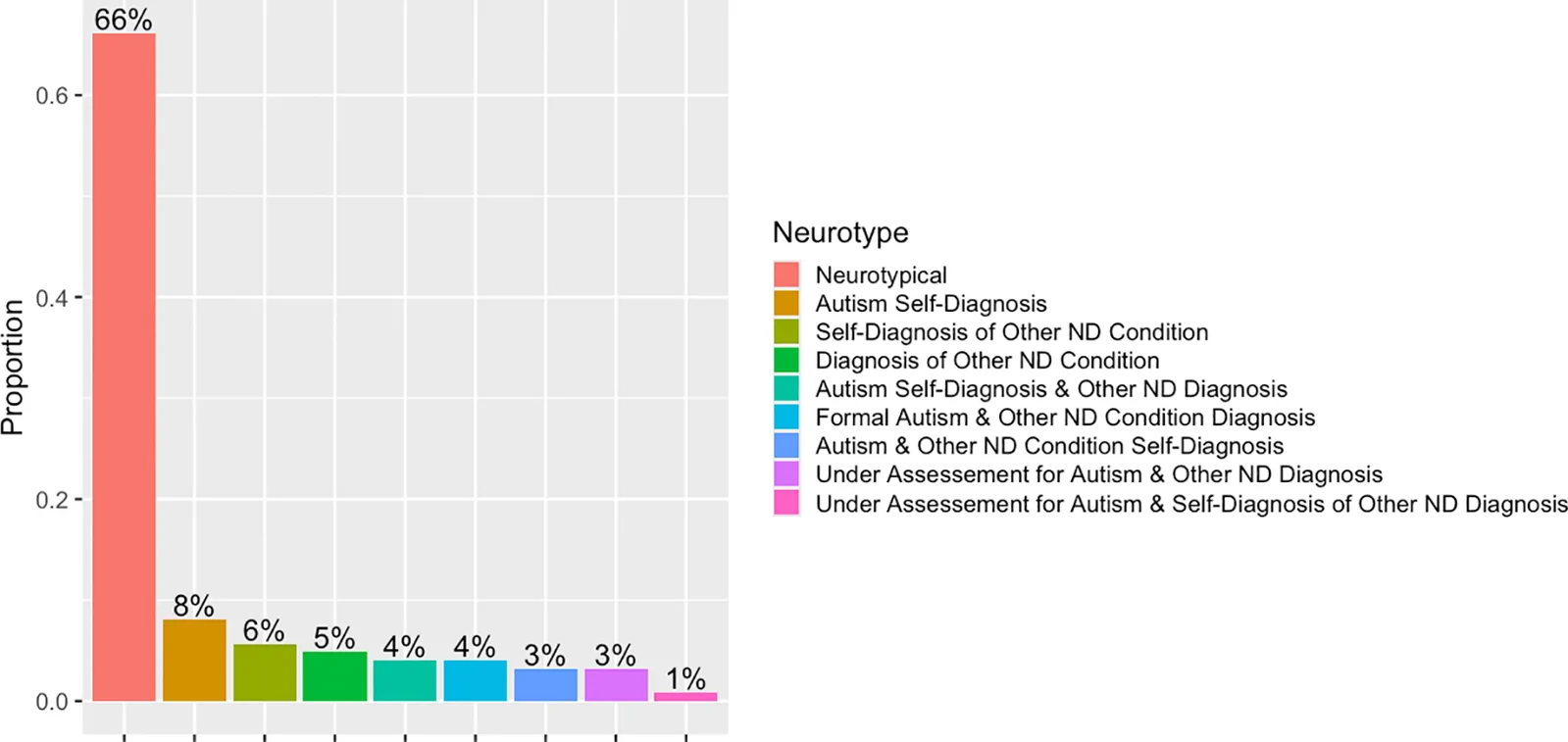
Distribution of neurotype categories among survey respondents, illustrating overlap between self-reported autism status (formal diagnosis, undergoing diagnostic assessment, or self-identified autism) and responses to the question regarding other developmental conditions.

### Association between professional categories and neurodiversity (Chi-Square Tests)

No statistically significant association was observed between neurotype and career category (χ ^2^(3) = 5.73, p = .16, Cramér’s V = .21). Although the observed effect size suggested a small-to-moderate association, the sensitivity analysis indicated that the study was powered to detect effects of approximately V = .31 or larger. Consequently, smaller differences in career distribution cannot be ruled out. Although the overall association was not statistically significant, the largest standardized residuals were observed for the early-career researcher and senior researcher categories (see Figure 2 and Table 2), with neurodivergent researchers appearing more frequently among early-career researchers and less frequently among senior researchers.

**Figure 2.**
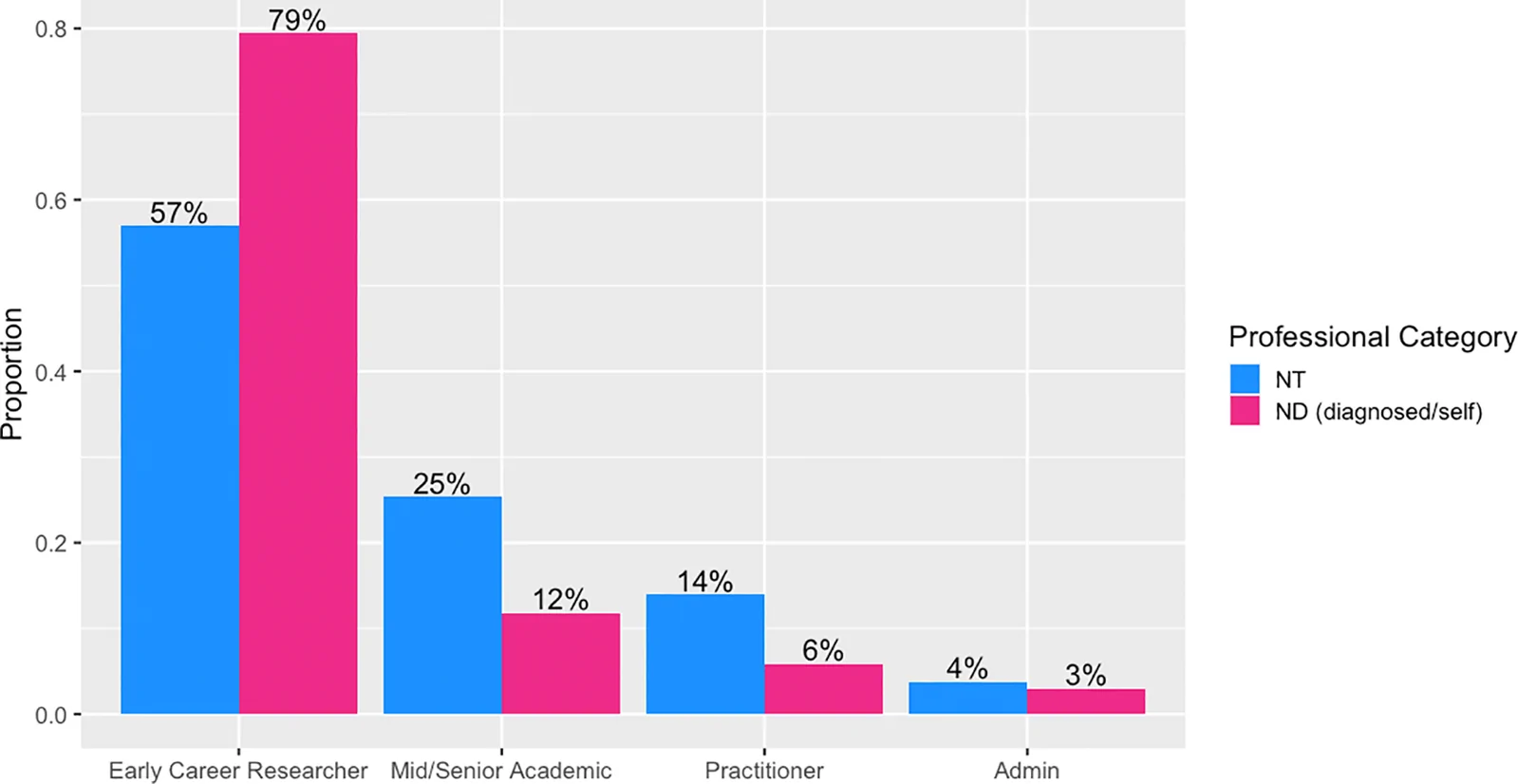
Graphical representation of professional category percentage distribution by neurotype.

**Table 2.** Standardized residuals for the chi-square analysis of neurotype by career pathway.

	Standardized Residual	
	Neurotypical	Neurodivergent
Admin	0.25	−0.25
Early Career Researcher	−2.36	2.36
Mid-Senior Academic	1.67	−1.67
Practitioner	1.12	−1.27

## Relationship with mental health diagnoses

The logistic regression included 105 participants with complete data on neurotype, age, gender and lifetime mental health diagnosis. Neurodivergence was positively associated with lifetime mental health diagnosis (Coef. = 1.67, SE = 0.48, Z-Value = 3.45, P-value = 0.0005; see Table 3 and Figure 3). Mental health diagnoses were also significantly more frequent in females (Coef. = 1.64, SE = 0.70, Z-Value = 2.34, P-value = 0.01). Age did not influence mental health diagnoses (Coef. = 0.004, SE = 0.02, Z-Value = 0.22, P-value = 0.82).

**Table 3.** Complete results of the multiple logistic regression, with formal mental health diagnosis as dependent variable.

	Estimate	SE	Z-Value	P-Value
Intercept	−2.73	1.13	−2.42	0.01
Neurodivergence (Diagnosed/Self )	1.67	0.48	3.45	0.0005*
Age	0.004	0.02	0.22	0.82
Gender (Woman)	1.64	0.70	2.34	0.01*

**Figure 3.**
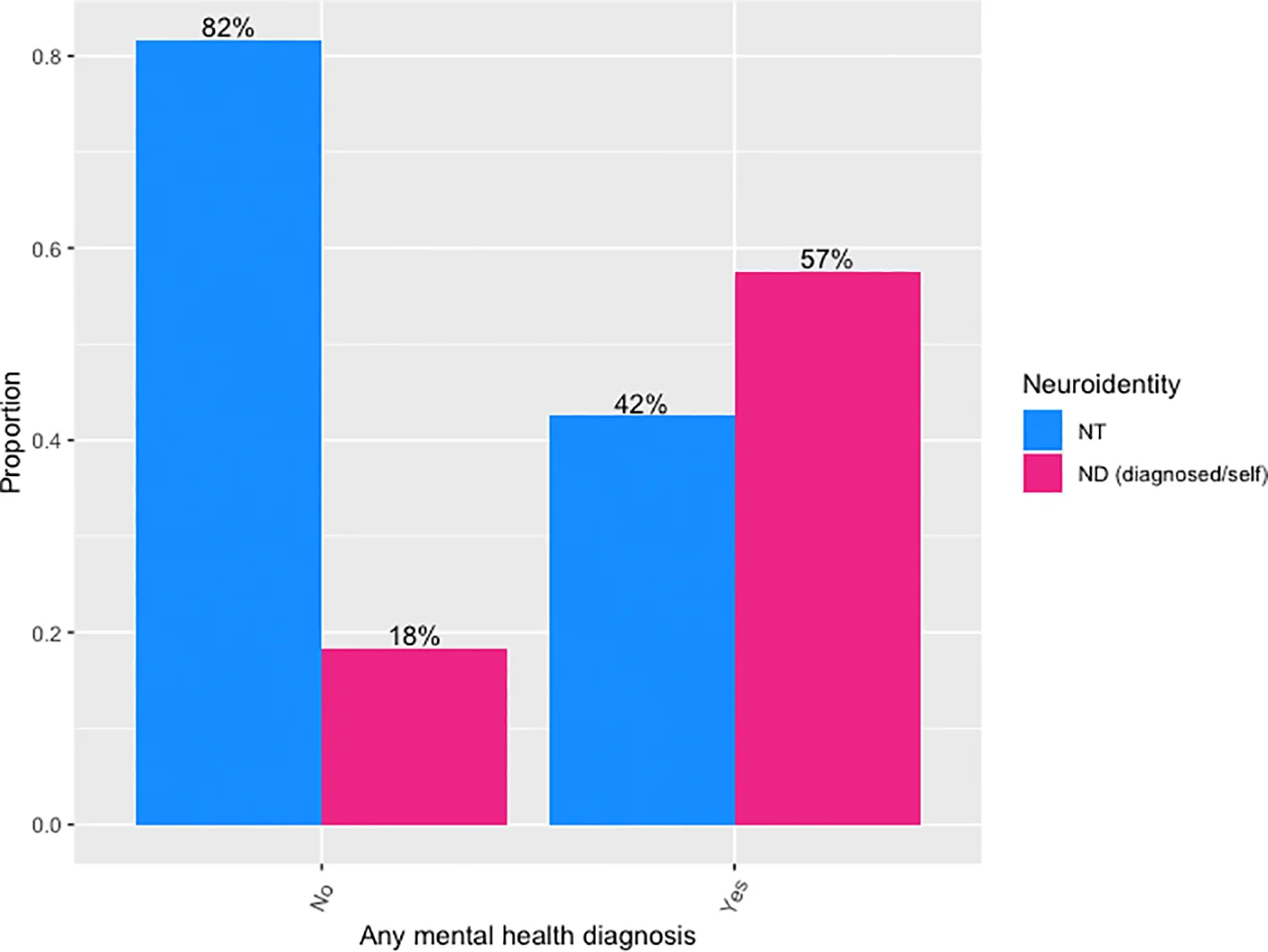
Graphical representation of the distribution of mental health diagnosis by neurotype.

To assess the robustness of the association between neurotype and lifetime mental health diagnosis, we conducted a Bayesian logistic regression including neurotype, age, and gender. The model showed satisfactory convergence (all R ^ = 1.00, ESS > 1000; see Table 4), good posterior predictive performance (see Figure 4). Consistent with the frequentist analysis, neurodivergence was positively associated with lifetime mental health diagnosis, with the 95% credible interval excluding zero ( Table 4).

**Table 4.** Complete results of the Bayesian multiple logistic regression, with formal mental health diagnosis as dependent variable.

	Estimate	Lower Bound 95%	Higher Bound 95%	Bulk ESS	Tail ESS	R ^
Intercept	0.05	<0.001	0.51	6058.83	5462.33	1.00
Neurodivergence (Diagnosed/Self )	5.67	2.23	14.96	6577.25	5637.73	1.00
Age	1.00	0.96	1.05	7976.00	5470.85	1.00
Gender (Woman)	6.14	1.59	30.43	6855.95	4748.20	1.00

**Figure 4.**
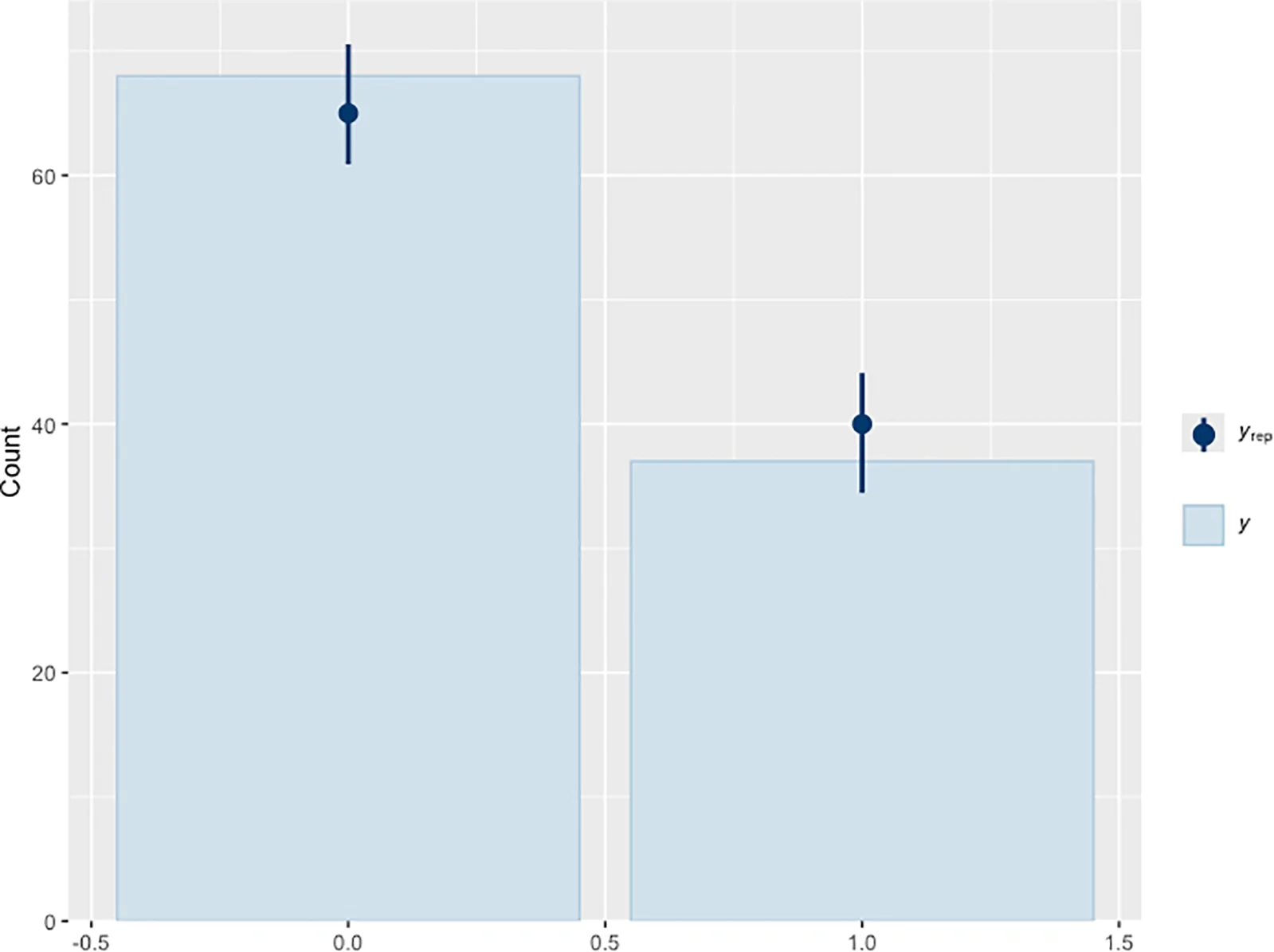
The observed outcome (blue bar) falls within the posterior predictive distribution (error bars), indicating good model fit.

## Discussion

The primary goal of this study was to investigate the presence of neurodivergent researchers within the research consortium. Additionally, we explored the distribution of neurodivergent researchers across career stages and examined the association between neurotype and self-reported lifetime mental health diagnoses.

Formal autism diagnoses were reported by 4% of respondents, with an additional 4% reporting that they were undergoing assessment and 15% self-identifying as autistic. Overall, neurodivergent researchers were represented across career stages within the consortium, although no statistically significant differences in distribution were observed.

The proportion of respondents reporting a formal autism diagnosis (4%) was higher than published estimates of autism prevalence in the general population (e.g., 0.6%, 95% CI: 0.1–1; Salari *et al.*, 2022). However, this comparison should be interpreted cautiously, as the present study was exploratory, based on a voluntary survey, and conducted within a specialised autism research consortium rather than a representative population sample. The relatively high proportion of respondents who self-identified as autistic without a formal diagnosis may reflect several factors, including increased awareness of autism within an autism research consortium, barriers to adult diagnosis ( Ardeleanu *et al.*, 2025), and the predominance of women in the sample, given the well-documented underdiagnosis of autism in females ( Kreiser & White, 2014).

Although neurodivergent researchers were represented across career stages, we did not observe statistically significant differences in their distribution within the consortium. This finding should be interpreted cautiously. The study was exploratory, and the number of neurodivergent researchers, particularly those with a formal autism diagnosis, was relatively small, limiting the ability to detect more subtle differences in career distribution.

Neurodivergence was associated with increased odds of reporting a lifetime mental health diagnosis, even after adjustment for age and gender. This finding is consistent with previous literature demonstrating elevated rates of anxiety, depression, and other mental health conditions among neurodivergent individuals ( Lai *et al.*, 2019; Lever & Geurts, 2016). Nevertheless, it is noteworthy that the prevalence of lifetime mental health diagnoses was relatively high across the sample as a whole, including among neurotypical researchers. This likely reflects the demanding nature of academic research careers, although the present study was not designed to investigate occupational contributors to mental health.

The presence of neurodivergent researchers within the consortium highlights an often overlooked dimension of participatory research. Participatory models have rightly emphasized the involvement of autistic community members and public representatives. However, our findings suggest that lived experience may already exist within the research workforce itself. Nevertheless, neurodivergent researchers occupy a dual position: they contribute both professional expertise and, for some, lived experience of neurodivergence ( Abubakare, 2022; Botha, 2021). This perspective may enrich research by helping to identify assumptions, improve study design, and broaden interpretation. At the same time, neurodivergent researchers remain members of the academic community and therefore occupy a different position from community representatives participating through formal involvement structures. Their lived experience should therefore be recognising and viewed as complementary to, rather than as a replacement for, established participatory practices.

We also observed a relatively low rate of disclosure of neurodivergent identities (see Table S3 in Supplementary Material 2), particularly among researchers who self-identified as autistic without a formal diagnosis. The survey was not designed to investigate reasons for disclosure, and these may include uncertainty around diagnosis, privacy preferences, workplace culture, or concerns around stigma. Previous research has highlighted that neurodivergent professionals may experience apprehension about disclosing their identities within academic and professional settings ( Abubakare, 2022; Botha, 2021; Jacques *et al.*, 2022). Although AIMS-2-TRIALS has implemented several initiative to promote neuro-affirmative research practices and collaboration with the autistic community, our findings suggest that further work may be beneficial to ensure that researchers feel able to disclose their identities and request support where appropriate.

Although the survey was primarily quantitative, participants also responded to two open-ended questions regarding current and future priorities for autism research. These responses were not intended for formal qualitative analysis, but illustrate the diversity of perspectives held by neurodivergent researchers within the consortium (see Table S4 in Supplementary Material 2).

### Limitations

A limitation of this study is that it was not possible to determine a precise response rate. The survey was distributed through consortium mailing lists; however, AIMS-2-TRIALS does not maintain a fixed census of active researchers. Membership of research teams changes over time, particularly among early-career researchers and project staff, and individuals may continue to contribute to consortium analyses after moving to other institutions or positions. Consequently, the total number of researchers eligible to participate at the time of survey distribution could not be established reliably. The findings should therefore be interpreted as reflecting the characteristics of survey respondents rather than a definitive estimate of the entire consortium workforce. In addition, the absolute number of autistic individuals in the sample was relatively small, warranting caution when interpreting subgroup analyses and generalising the findings.

An additional limitation concerns the scope and framing of the survey questions. To minimise participant burden and preserve anonymity, several questions used broad categories rather than more detailed information (e.g., predefined examples and self-report categories for mental health conditions and neurodevelopmental differences). This may have obscured heterogeneity within both neurodivergent and neurotypical groups, and may have influenced responses towards the conditions explicitly listed.

To reduce concerns regarding identifiability, responses were anonymous and participants were informed that all data would remain confidential. In addition, all questions were optional, allowing participants to omit information they considered potentially identifying. These measures were implemented to facilitate participation from researchers who might otherwise have concerns about disclosure. Nevertheless, it remains possible that researchers who were less comfortable disclosing aspects of their identity chose not to participate or omitted particular responses. Furthermore, the participant information sheet described the study as focusing on neurodivergence and well-being. This framing may also have influenced participation and response patterns, potentially increasing engagement among individuals with a particular interest in neurodiversity or personal experience of mental health difficulties. As a result, self-selection and response biases cannot be ruled out.

## Conclusions

This exploratory survey provides an initial description of the presence of neurodivergent researchers within a large European autism research consortium. Neurodivergent researchers were represented across a range of career stages, and neurodivergence was associated with increased odds of reporting a lifetime mental health diagnosis. Together, these findings highlight that lived experience of neurodivergence is present within the autism research workforce and should be recognised alongside established participatory approaches involving community representatives.

The present findings suggest several priorities for future work. First, replication across other autism and neuroscience research settings is needed to establish whether similar patterns are observed beyond AIMS-2-TRIALS. Second, longitudinal studies could investigate career progression and disclosure over time, while qualitative research could explore workplace experiences, support needs, and the relationship between professional and lived expertise among neurodivergent researchers. Finally, research organisations adopting participatory approaches may benefit from considering how neurodivergent researchers within the workforce can contribute alongside community representatives, while ensuring that appropriate support and opportunities for disclosure are available.

## Declarations

Several authors are current or former members of the AIMS-2-TRIALS consortium. The present study was conducted independently and was not commissioned by the consortium.

## Ethics and consent

Ethical approval for this study was obtained from the Departmental Ethics Committee of the Department of Psychological Sciences at Birkbeck College, University of London on 26/07/2021 (reference number: 2021091).

Members of AIMS-2-TRIALS received the invitation and link providing access to the survey via email from the AIMS-2-TRIALS Communication Team in January 2022. Comprehensive details regarding the study aims and procedures were provided in both the email and the study information sheet displayed after clicking the link.

Participants could proceed to the consent at the bottom of the information screen.

Participants provided written consent by confirming that:

• They understood the details of the study as explained to them and willingly consented to take part.
• They were aware that they could ask further questions to the study investigator via email at any time.
• They understood that their participation would remain anonymous, and all information provided would be used solely for the purposes of the study.
• They could decline to answer specific questions and withdraw their data at any time before submitting the questionnaire, after which the anonymized data could no longer be identified.
• All information provided would be kept confidential, and no personally identifiable data (e.g., name, date of birth) would be collected.
• Anonymous responses would be stored securely and accessed only by authorized researchers.
• The results of the study would be written up for a project/thesis and presented at conferences and in academic journals. Any individual data presented would be completely anonymous, with no means of identifying participants.
• They were 18 years or older.

After confirming consent, participants could proceed to the survey webpage and progress between questions.

## Materials

Supplementary Material 1, 2.

Data are available under the terms of the Creative Commons Attribution 4.0 International license (CC-BY 4.0).

All the materials (including the information sheet, consent form and survey questions), analysis code and extended data have been made available on a public access repository at the following link: https://doi.org/10.17605/OSF.IO/7PBVR.

Owing to the sensitive nature of the data and the risk of deductive disclosure within a relatively small research community, the dataset is not publicly available. Requests for access to the data for the purpose of reproducing the analyses may be directed to the corresponding author and will be considered subject to ethical and data protection requirements. This approach aligns with the specific security and privacy requirements outlined in the participants’ consent for this study (available along with all study materials at the link above), as approved by the Departmental Ethics Committee of the Department of Psychological Sciences at Birkbeck College, University of London. Additionally, it is supported by evidence indicating that autistic individuals and their families prefer curated over open data-sharing (see Ashworth *et al.*, 2021; Begum Ali *et al.*, 2024; Hobson *et al.*, 2023).

## Data Availability

Del Bianco, T. (2026, July 2). Neurodivergent Researchers in Autism Research: A Survey within the AIMS-2-TRIALS Consortium. https://doi.org/10.17605/OSF.IO/7PBVR The project contains the following underlying data: Analysis Scripts. Ethics.
