# Supplementary material for: Neurodivergent Researchers in Autism Research: A Survey within the AIMS-2-TRIALS Consortium
Source: Open Res Eur. 2026 Jul 9;5:120. Originally published 2025 May 6. [Version 2] doi: 10.12688/openreseurope.19394.2 (PMC13560805; doi:10.12688/openreseurope.19394.2)
Supplement: Peer review history

# Reviewer report: Reviewer response for version 2

**DOI:** 10.21956/openreseurope.26533.r77268

**Published:** 2026-08-04

**Authors:** Lauren Lestremau Allen

**Affiliations:** 1 SUNY Empire State University, New York, NY, USA

Thanks to the authors for investigating this area by examining the extent to which Autistic and Neurodivergent researchers are represented in the membership of the targeted research consortium. The work clearly and concisely offers a purpose, outlines methodology, and presents results and implications. It engages with relevant literature and offers detailed materials and methodology to allow for replication. Although materials are included, data are not available due to the risk for identification as a result of the small community. The conclusions are aligned to the provided results and the recommendations for future research were meaningful. The authors concluded higher proportions of Autistic and Neurodivergent individuals were represented in the sample than in the population, with substantial participants undergoing assessment or self-identifying. The revisions made in the earlier round(s) were supportive based on the summary provided of those changes.

If applicable, is the statistical analysis and its interpretation appropriate?

I cannot comment. A qualified statistician is required.

Is the study design appropriate and is the work technically sound?

Yes

Is the work clearly and accurately presented and does it engage with the current literature?

Yes

Are the conclusions drawn adequately supported by the results?

Yes

Are sufficient details of methods and analysis provided to allow replication by others?

Yes

Are all the source data and materials underlying the results available?

No

Reviewer Expertise:

Neurodiversity and disability as diversity, neuro-affirming and neuro-inclusive approaches particularly in higher education and P-12 education, Universal Design, and community-informed research

I confirm that I have read this submission and believe that I have an appropriate level of expertise to confirm that it is of an acceptable scientific standard.

# Reviewer report: Reviewer response for version 2

**DOI:** 10.21956/openreseurope.26533.r77175

**Published:** 2026-07-16

**Authors:** Emma Wilson

**Affiliations:** 1 University of Edinburgh, Edinburgh, England, UK

I'd like to thank the authors for taking the time to read and respond to my comments. I have now read through the responses and the revised version of the manuscript and am happy that my comments have been addressed.

If applicable, is the statistical analysis and its interpretation appropriate?

I cannot comment. A qualified statistician is required.

Is the study design appropriate and is the work technically sound?

Yes

Is the work clearly and accurately presented and does it engage with the current literature?

Yes

Are the conclusions drawn adequately supported by the results?

Yes

Are sufficient details of methods and analysis provided to allow replication by others?

Yes

Are all the source data and materials underlying the results available?

Partly

Reviewer Expertise:

Autism research, open research practices, research support, and research culture

I confirm that I have read this submission and believe that I have an appropriate level of expertise to confirm that it is of an acceptable scientific standard.

# Reviewer report: Reviewer response for version 1

**DOI:** 10.21956/openreseurope.20988.r60174

**Published:** 2025-09-30

**Authors:** Emma Wilson

**Affiliations:** 1 University of Edinburgh, Edinburgh, England, UK

Thank you for the opportunity to review this work. Overall, I thought it was a highly interesting and important study. I’ve added some comments below to improve clarity in places and also to strength the call to action of the paper.

 Abstract / Plain Summary

- Perhaps the work could benefit from a sentence describing the overarching aims of the AIMS-2-TRIALS Autism Research Consortium, to provide some context?

Introduction

- Definitions of neurodiversity and neurodivergent in the first paragraph of the introduction would be helpful to set the scene
- The references are good but I think you could make it more explicit in the text that autistic people have historically been excluded from autistic research and what impact that has had on autism research, including why the Neurodiversity Paradigm changes that narrative
- In the sentence beginning “Since its inception in 2018…”, are you able to include any information on how the consortium has been perceived by the autistic community as a marker for impact (I’m conscious that publication and citation counts shouldn’t be the only impact markers used)?
- Can you clarify what “promoting shared practices and data” refers to? My assumption is that data might mean data sharing – if so, what kind of data? And I’m not sure what shared practices means.
- It may be my misunderstanding, but I’m curious about the use of the term “non-neurodivergent” as opposed to the more commonly used term “neurotypical” in the paragraph beginning “Over the duration of this consortium…”

Methods

- Missing the words “are” and “and “from the sentence “Our Methods, Results and Limitations sections and supplementary material [are] available online (see Data Availability) [and] follow best practices to ensure reproducibility by providing sufficient detail on materials and methods…”
- The word “a” should be removed from the sentence “Recruitment was carried out in January 2022 through the AIMS-2-TRIALS internal mailing list, with [a] four emails…”
- Referring to the sentence “Members of AIMS-2-TRIALS received the invitation and link providing access to the survey via email from the AIMS-2-TRIALS Communication Team in January 2022”, where all four emails sent in January 2022? If not, can you provide the timeline?
- The word “samples” should be replaced with “sample” in the sentence “To increase the [samples] sizes at the level of individual categories”

Results

- Some of the colour contrast in the pie chart figures (e.g., colour of text compared to background colour) is low and makes the text hard to read. I recommend the WebAim Contrast Checker ( https://webaim.org/resources/contrastchecker/) to make sure there is adequate colour contract between text and background colour.
- Referring to the sentence “76.61% responded that they are not autistic, under assessment or think they are/might be autistic” the wording here tripped me up because it made it seem like these respondents thought they are/might be autistic rather than didn’t think they were. Perhaps could be reworded?
- Referring to the sentence “All self-detected respondents left this question blank” does this mean that all self-detected respondents did not say whether they had disclosed to their line manager? If so, could this be made clearer?
- Referring to respondent gender, did any respondents identify as non-binary? Could this be clarified in the text? I can see from the supplementary materials that non-binary was an option, but this doesn’t come across in the text, so it’s unclear if no one responded as such or it simply wasn’t an option.

Discussion

- It might be helpful to have a separate action items or recommendations section to pull out some of the suggestions from the discussion section, as currently I think there get a little lost on the text.

Data availability

- Very minor and not obligatory at all but it might be worthwhile to assign a DOI to your OSF project
- It may be easier to navigate the supplementary material if each material (e.g., survey questions, survey results, contributions) is its own file. Additionally, survey result may be better suited to a spreadsheet/CSV file format than PDF.
- To make the analysis code more reproducible, it may be helpful to add an anonymised dataset (only if this can be shared).
- In the survey results, can you place “prefer not to say” at the end of each of the options, rather than alphabetically, so it’s easier to see?
- It may be easier to navigate the OSF project if you include a README (could be part of the project wiki text) that specifies the folder structure and a brief description of each files’ contents.

If applicable, is the statistical analysis and its interpretation appropriate?

I cannot comment. A qualified statistician is required.

Is the study design appropriate and is the work technically sound?

Yes

Is the work clearly and accurately presented and does it engage with the current literature?

Yes

Are the conclusions drawn adequately supported by the results?

Yes

Are sufficient details of methods and analysis provided to allow replication by others?

Yes

Are all the source data and materials underlying the results available?

Partly

Reviewer Expertise:

Autism research, open research practices, research support, and research culture

I confirm that I have read this submission and believe that I have an appropriate level of expertise to confirm that it is of an acceptable scientific standard, however I have significant reservations, as outlined above.

# Response

**Published:** 2026-07-02

**Authors:** Teresa Del Bianco

**Affiliations:** School of Social Science and Professions, London Metropolitan University School of Social Sciences and Professions, London, England, UK

Thank you for taking the time to thorougly review our work. Please find our responses and outline of changes to the manuscript below: **Abstract / Plain Summary**

- Perhaps the work could benefit from a sentence describing the overarching aims of the AIMS-2-TRIALS Autism Research Consortium, to provide some context?
  - Thank you for this suggestion. We have expanded the description of AIMS-2-TRIALS in the Introduction to provide additional context for readers unfamiliar with the consortium. Specifically, we now describe its multidisciplinary composition, overarching aims to improve understanding of autism heterogeneity and support the development of personalised interventions, and its inclusion of autistic public representatives (A-Reps) as part of its participatory research approach (last paragraph of page 4).

**Introduction**

- Definitions of neurodiversity and neurodivergent in the first paragraph of the introduction would be helpful to set the scene
  - Thank you for this suggestion. We have added definitions of both neurodiversity and neurodivergence to the opening paragraph of the Introduction and clarified their relationship to the Neurodiversity Paradigm.
- The references are good but I think you could make it more explicit in the text that autistic people have historically been excluded from autistic research and what impact that has had on autism research, including why the Neurodiversity Paradigm changes that narrative
  - Thank you for this helpful suggestion. We have revised the Introduction to make this historical context more explicit. Specifically, from page 5: “Historically, autism research has been conducted by researchers, with autistic people having limited involvement in determining research priorities, study design and interpretation (…) The Neurodiversity Paradigm has further contributed to this shift by emphasising neurodivergent lived experience as a valuable source of expertise, rather than viewing autism solely through a deficit-based lens”.
- In the sentence beginning “Since its inception in 2018…”, are you able to include any information on how the consortium has been perceived by the autistic community as a marker for impact (I’m conscious that publication and citation counts shouldn’t be the only impact markers used)?
  - We agree that publication and citation metrics do not fully capture impact. However, we were unable to identify published evaluations of the consortium's impact from the perspective of autistic communities. Rather than speculate about community perceptions, we have revised the text to focus on the consortium's scientific outputs and its participatory structures, including the involvement of autistic representatives (A-Reps).
- Can you clarify what “promoting shared practices and data” refers to? My assumption is that data might mean data sharing – if so, what kind of data? And I’m not sure what shared practices means.
  - Thank you for this observation. We agree that the wording was unclear. The phrase referred to collaboration and data sharing between consortium partners, including the harmonisation and pooling of research data across participating sites. However, as this detail was not essential to the rationale for the present study, we have removed the sentence and simplified the description of the consortium.
- It may be my misunderstanding, but I’m curious about the use of the term “non-neurodivergent” as opposed to the more commonly used term “neurotypical” in the paragraph beginning “Over the duration of this consortium…”
  - Thank you for highlighting this point. We agree that "neurotypical" is the more widely used term in this context. To improve clarity and consistency with the broader literature, we have replaced "non-neurodivergent" with "neurotypical" throughout the manuscript where appropriate.

**Methods**

- Missing the words “are” and “and “from the sentence “Our Methods, Results and Limitations sections and supplementary material [are] available online (see Data Availability) [and] follow best practices to ensure reproducibility by providing sufficient detail on materials and methods…”
  - Thank you for noticing this, it has been corrected.
- The word “a” should be removed from the sentence “Recruitment was carried out in January 2022 through the AIMS-2-TRIALS internal mailing list, with [a] four emails…”
  - Thank you for noticing this, it has been corrected.
- Referring to the sentence “Members of AIMS-2-TRIALS received the invitation and link providing access to the survey via email from the AIMS-2-TRIALS Communication Team in January 2022”, where all four emails sent in January 2022? If not, can you provide the timeline?
  - Thank you for highlighting this ambiguity. The original wording may have implied that all four emails were distributed in January 2022. While recruitment commenced in January 2022, the precise dates of the subsequent reminder emails are no longer available to the authors. We have therefore revised the manuscript to clarify that recruitment began in January 2022 and that reminder emails were distributed during the recruitment period.
- The word “samples” should be replaced with “sample” in the sentence “To increase the [samples] sizes at the level of individual categories”
  - Thank you for noticing this, it has been corrected.

**Results**

- Some of the colour contrast in the pie chart figures (e.g., colour of text compared to background colour) is low and makes the text hard to read. I recommend the WebAim Contrast Checker (https://webaim.org/resources/contrastchecker/) to make sure there is adequate colour contract between text and background colour.
  - Thank you for this helpful suggestion. We have revised the figures for better contrast and clarity.
- Referring to the sentence “76.61% responded that they are not autistic, under assessment or think they are/might be autistic” the wording here tripped me up because it made it seem like these respondents thought they are/might be autistic rather than didn’t think they were. Perhaps could be reworded?
  - Thank you for raising this, we have substantially reviewed this section for clarity and readability.
- Referring to the sentence “All self-detected respondents left this question blank” does this mean that all self-detected respondents did not say whether they had disclosed to their line manager? If so, could this be made clearer?
  - Thank you for this comment. We have now reworded this sentence for clarity and moved it to the Discussion: “We also observed a relatively low rate of disclosure of neurodivergent identities (see Table S3 of the Supplementary Material), particularly among researchers who self-identified as autistic without a formal diagnosis.”
- Referring to respondent gender, did any respondents identify as non-binary? Could this be clarified in the text? I can see from the supplementary materials that non-binary was an option, but this doesn’t come across in the text, so it’s unclear if no one responded as such or it simply wasn’t an option.
  - Participants could indicate their gender as male, female, non-binary, or self-describe. Given the very small number of respondents selecting non-binary or self-described gender identities, these categories are not reported separately in the main text in order to reduce the risk of deductive disclosure, however, we have included it in Table 1. Where gender is reported descriptively or included in analyses, categories were collapsed into female versus all other responses.

**Discussion**

- It might be helpful to have a separate action items or recommendations section to pull out some of the suggestions from the discussion section, as currently I think there get a little lost on the text.
  - Thank you for this suggestion. We have now included a separate paragraph at the end of the Conclusions: “The present findings suggest several priorities for future work. First, replication across other autism and neuroscience research settings is needed to establish whether similar patterns are observed beyond AIMS-2-TRIALS. Second, longitudinal studies could investigate career progression and disclosure over time, while qualitative research could explore workplace experiences, support needs, and the relationship between professional and lived expertise among neurodivergent researchers. Finally, research organisations adopting participatory approaches may benefit from considering how neurodivergent researchers within the workforce can contribute alongside community representatives, while ensuring that appropriate support and opportunities for disclosure are available.”

**Data availability**

- Very minor and not obligatory at all but it might be worthwhile to assign a DOI to your OSF project
  - Thank you for this suggestion. We have now created a DOI for the OSF project
- It may be easier to navigate the supplementary material if each material (e.g., survey questions, survey results, contributions) is its own file. Additionally, survey result may be better suited to a spreadsheet/CSV file format than PDF.
  - Thank you for your suggestion. We have now converted the supplementary material to an excel spreadsheet.
- To make the analysis code more reproducible, it may be helpful to add an anonymised dataset (only if this can be shared).
  - Thank you for this suggestion. Due to the sensitive nature of the data and the relatively small and identifiable research community from which participants were recruited, we are unable to make the dataset publicly available, as complete anonymisation cannot be guaranteed. However, to promote transparency and reproducibility, we have made the analysis code available. Researchers interested in accessing the dataset for the purpose of reproducing the analyses may contact the corresponding author, and requests will be considered in accordance with the participants' consent and applicable ethical and data protection requirements (see Data Availability statement).
- In the survey results, can you place “prefer not to say” at the end of each of the options, rather than alphabetically, so it’s easier to see?
  - Thank you for your suggestion, we have moved the prefer not to say option to the end of each option.
- It may be easier to navigate the OSF project if you include a README (could be part of the project wiki text) that specifies the folder structure and a brief description of each files’ contents.
  - Thank you for this suggestion, we have now created a READ ME front page for the OSF project and in the 'Analysis Scripts' folder.

# Reviewer report: Reviewer response for version 1

**DOI:** 10.21956/openreseurope.20988.r54311

**Published:** 2025-06-13

**Authors:** Lucy H Waldren, Luca D Hargitai, Olujolagbe Layinka

**Affiliations:** 1 University of Bath, Bath, UK

Thank you for the opportunity to review *Examining Neurodiversity and Inclusion in Neuroscience Research Networks: A Case Study of the AIMS-2-TRIALS Autism Research Consortium*. This paper presents an exploratory investigation into the prevalence and distribution of neurodivergent researchers within the AIMS-2-TRIALS Autism Research Consortium. Whilst helpful, we believe this works would benefit from substantial revisions to improve its transparency, rigor, and accuracy.

**Introduction**

- Considering the broad scope of research published in Open Research Europe, readers may benefit from a description and definition of neurodiversity.
- Please could the authors clarify whether the ‘shared practices and data’ resulting from the consortium refers to internal collaborations or published openly accessible datasets, as the examples currently provided are not associated with open data?
- Please could the authors provide specific examples of when ‘principal investigators have [prioritized] the recruitment of professionals who openly identify as autistic’?
- The final paragraph of the Introduction needs to be rephrased to significantly reduce the importance and impact that can be drawn from this work. Indeed, a running issue in this paper (that is reflected in our later comments) was that findings and interpretations were continually drawn beyond what can accurately be determined from the current results – that is – an estimation of prevalence and distribution of neurodivergent researchers within the AIMS-2-TRIALS Autism Research Consortium.

**Methods **

- Please could the authors comment on whether this work was pre-registered, clarifying that this is an exploratory investigation if appropriate.
- This work would benefit from further details concerning sample size. First, without knowing how many people were invited to complete the survey, it is difficult to ascertain the representativeness of the current sample compared to the wider AIMS-2-TRIALS Consortium. Second, readers would benefit from sensitivity power analyses, to gauge the generalisability of the current results. Third, considering questions were optional, it is important to clarify how you handled missing data.
- We strongly encourage the authors to reflect and comment on the limitations and potential biases within their survey questions. For example, questions on mental health (e.g., anxiety and depression) and other neurodevelopmental conditions (e.g., ADHD or dyslexia) both limit future interpretations by not collecting more nuanced insight whilst also potentially biasing responses towards only the example conditions listed. Furthermore, the information sheet highlighted that this work was interested in exploring neurodivergence and wellbeing – please could the authors comment on how they think this may have primed responses across both neurodivergent and neurotypical participants?

**Results**

- The authors should specify that multiple *logistic *regression models were run and report null results in addition to significant outcomes in the Main Text. Indeed, given the large quantity of null results, the current findings would be bolstered by the inclusion of supplementary Bayesian analyses that can help to quantify the evidence for the null vs alternative hypothesis.
- We would recommend moving the demographics table from the Supplementary Material to the Main Text, as this information is very helpful for interpreting the wider results.
- The clarity of all figures could be improved. Specifically, we recommend using a more distinct colour palette in Figure 1 and including a y-axis label and figure note for Figures 2 and 3 highlighting that this is percentage per group, to avoid incorrect or misleading interpretations.

**Discussion**

 The Discussion needs to be substantially re-written to more accurately reflect the conclusions and interpretations that are permissible from the current results:

- The researchers do not ask any questions about the lived experiences of the participants or their perceptions of inclusivity and support. As such, numerous statements throughout the Discussion need to be rephrased and diluted as they cannot be interpreted from the current results.
- Non-disclosure does not inherently mean that participants are stigmatised. Participants may not have felt the need to disclose their diagnoses because they are already well-accommodated. Whilst we agree stigma is an important issue to consider, stigma was not explicitly measured and thus these assumptions cannot be drawn.
- Similarly, you say wider initiatives within the AIMS-2-TRIALS help to reduce stigma, however this was not assessed and thus cannot be known.
- When you discuss wider neurodevelopmental and mental health conditions, it needs to be made clear that these are only examples, as specific conditions were not accounted for in your methodology.
- The qualitative questions specifically focus on participants’ research trajectories and cannot be used to infer their personal sense of satisfaction and belonging.
- Please could the researchers comment on the potential impact of age on the higher prevalence of neurodivergent ECRs, rather than solely focussing on the (untested) link between neurodivergence and lack of career progression support?

**General **

- In light of the above, it may be apt for the authors to re-phrase their title, to more accurately reflect the current research.
- It should be noted as a potential conflict of interest that the authors are funded by the AIMS-2-TRIALS they are currently investigating.
- We encourage the authors to be consistent in their use of neurodiverse language, in line with current recommendations (e.g., Bottema-Beutel et al., 2021).

*Please note, all authors contributed equally to this review.

If applicable, is the statistical analysis and its interpretation appropriate?

Partly

Is the study design appropriate and is the work technically sound?

Partly

Is the work clearly and accurately presented and does it engage with the current literature?

Partly

Are the conclusions drawn adequately supported by the results?

No

Are sufficient details of methods and analysis provided to allow replication by others?

Yes

Are all the source data and materials underlying the results available?

Yes

Reviewer Expertise:

Neurodevelopmental Conditions, Quantitative Methods.

We confirm that we have read this submission and believe that we have an appropriate level of expertise to confirm that it is of an acceptable scientific standard, however we have significant reservations, as outlined above.

## References

[1] : Avoiding Ableist Language: Suggestions for Autism Researchers. *Autism in Adulthood* .2021;3(1) : 10.1089/aut.2020.0014 18-29 10.1089/aut.2020.0014 PMC899288836601265

# Response

**Published:** 2026-07-02

**Authors:** Teresa Del Bianco

**Affiliations:** School of Social Science and Professions, London Metropolitan University School of Social Sciences and Professions, London, England, UK

Thank you to the reviewers for taking the time to thoroughly review our work. Please find below a point-by-point response outlining the changes to the manuscript based on the feedback. **Introduction** 1) *Considering the broad scope of research published in Open Research Europe, readers may benefit from a description and definition of neurodiversity.* Thank you for this suggestion. We have now added definitions of both neurodiversity and neurodivergence in the opening paragraph of the Introduction. Specifically, we clarify that neurodiversity refers to the diversity of human neurocognitive functioning across populations, while neurodivergent refers to individuals whose neurological development or functioning differs from societal norms, including autistic people and those with other neurodevelopmental conditions. We have also linked these definitions to the Neurodiversity Paradigm to provide additional context for readers. 2) *Please could the authors clarify whether the ‘shared practices and data’ resulting from the consortium refers to internal collaborations or published openly accessible datasets, as the examples currently provided are not associated with open data?* Thank you for this observation. We agree that the wording was unclear. The phrase referred to collaboration and data sharing between consortium partners, including the harmonisation and pooling of research data across participating sites. However, as this detail was not essential to the rationale for the present study, we have removed the sentence and simplified the description of the consortium. 3) *Please could the authors provide specific examples of when ‘principal investigators have [prioritized] the recruitment of professionals who openly identify as autistic’?* Thank you for this observation. We agree that the original statement required stronger supporting evidence. Rather than provide anecdotal examples, we have revised the text to focus on documented discussions and initiatives aimed at supporting neurodivergent researchers, providing one example: “Individual researchers have shared their experience of neurodivergence with the public (Abubakare, 2022; Botha, 2021), and there is growing discussion within some research communities about actively supporting neurodivergent researchers (e.g., see for example the Neurodiversity and Participatory Research Advisory Group of the Respect4Neurodevelopment research community).” 4) *The final paragraph of the Introduction needs to be rephrased to significantly reduce the importance and impact that can be drawn from this work. Indeed, a running issue in this paper (that is reflected in our later comments) was that findings and interpretations were continually drawn beyond what can accurately be determined from the current results – that is – an estimation of prevalence and distribution of neurodivergent researchers within the AIMS-2-TRIALS Autism Research Consortium.* Thank you for this comment. We have now revised the last paragraph of the introduction as follows: “Here, we conducted a survey within the AIMS-2-TRIALS consortium to estimate the representation of neurodivergent researchers whilst maintaining participants’ privacy. Given the increasing emphasis on participatory approaches and the inclusion of neurodivergent perspectives in research (Hoekstra et al., 2018), understanding the composition of a research workforce represent and important, yet relatively underexplored, area of inquiry. The study aimed to describe prevalence and distribution of neurodivergence researchers within the consortium, and explore how neurotype relates to selected demographic and occupational characteristics, such as career stage, and self-reported mental health. As an exploratory investigation, the study was not designed to test specific hypotheses, but rather to provide an initial characterisation within this consortium and inform future discussions regarding diversity, inclusion and researcher well-being in autism research settings”. **Methods**

 5) *Please could the authors comment on whether this work was pre-registered, clarifying that this is an exploratory investigation if appropriate.* Thank you for this suggestion. We have now clarified both in the Introduction and Methods that this study was exploratory in nature and was not pre-registered. Specifically, from the last paragraph of the Introduction: “The study aimed to describe prevalence and distribution of neurodivergence researchers within the consortium, and explore how neurotype relates to selected demographic and occupational characteristics, such as career stage, and self-reported mental health. As an exploratory investigation, the study was not designed to test specific hypotheses, but rather to provide an initial characterisation within this consortium and inform future discussions regarding diversity, inclusion and researcher well-being in autism research settings.”, and from the Methods > Measures and Statistical Analysis: “The following analyses were exploratory in nature and were not pre-registered. Analyses were designed to provide an initial description of the prevalence and distribution of neurodivergent researchers within the consortium and to explore associations between neurotype and selected demographic, occupational, and mental health characteristics. Accordingly, all analyses should be interpreted as exploratory rather than confirmatory.” 6) *This work would benefit from further details concerning sample size. First, without knowing how many people were invited to complete the survey, it is difficult to ascertain the representativeness of the current sample compared to the wider AIMS-2-TRIALS Consortium. Second, readers would benefit from sensitivity power analyses, to gauge the generalisability of the current results. Third, considering questions were optional, it is important to clarify how you handled missing data.* Thank you for these useful comments. Please find below each of them addressed by theme. Sample size: We agree that the absence of a defined sampling frame limits conclusions regarding representativeness. Unfortunately, it was not possible to determine the exact number of eligible researchers at the time of survey distribution. AIMS-2-TRIALS does not maintain a fixed census of active researchers, and membership changes frequently due to staff turnover, particularly among ECRs and project staff. In addition, researchers may continue to contribute to consortium analyses after moving to other institutions or positions. We have now clarified this limitation in the Limitations, concluding that: “The findings should therefore be interpreted as reflecting the characteristics of survey respondents rather than a definitive estimate of the entire consortium workforce”.

Sensitivity analyses: Thank you for this helpful suggestion. We have now included a sensitivity power analysis for the chi-square test in the Methods section: “To assess the distribution of autistic and neurotypical researchers across career pathways and roles, we performed a Chi-Square Test of association in R (Posits Studio, 2025). To aid interpretation of the analysis, sensitivity power analyses were conducted in G*Power (Faul et al., 2009). With N = 124, df = 12, α = .05 and 80% power, the minimum detectable effect size was w = 0.37”. In addition, to assess the robustness of the logistic regression findings, we conducted a Bayesian logistic regression using the same model specification (see one of the points below).

Missing data: We have clarified how missing data were handled (see second paragraph of section ‘Measures and Statistical Analysis’). Optional questions were treated as missing values and were not imputed. Analyses were conducted using available-case data, with participants excluded only from analyses involving variables for which responses were missing. We also clarified that three free-text occupational responses were reclassified into existing categories based on the reported primary role. 7) *We strongly encourage the authors to reflect and comment on the limitations and potential biases within their survey questions. For example, questions on mental health (e.g., anxiety and depression) and other neurodevelopmental conditions (e.g., ADHD or dyslexia) both limit future interpretations by not collecting more nuanced insight whilst also potentially biasing responses towards only the example conditions listed. Furthermore, the information sheet highlighted that this work was interested in exploring neurodivergence and wellbeing – please could the authors comment on how they think this may have primed responses across both neurodivergent and neurotypical participants?* Thank you for this comment. We have expanded the Limitations section to reflect these points: “An additional limitation concerns the scope and framing of the survey questions. (…) This may have obscured heterogeneity within both neurodivergent and neurotypical groups, and may have influenced responses towards the conditions explicitly listed”. And: “the participant information sheet described the study as focusing on neurodivergence and well-being. This framing may also have influenced participation and response patterns, (…) As a result, self-selection and response biases cannot be ruled out.” **Results** 8) *The authors should specify that multiple logistic regression models were run and report null results in addition to significant outcomes in the Main Text. Indeed, given the large quantity of null results, the current findings would be bolstered by the inclusion of supplementary Bayesian analyses that can help to quantify the evidence for the null vs alternative hypothesis.* Thank you for this helpful suggestion. We have revised the regression analyses and Methods section to clearly specify that a multiple logistic regression model was fitted. To better align the analysis with the revised exploratory aims of the manuscript, the model was simplified to include the primary predictor of interest (neurotype) and the demographic covariates age and gender. We now report the regression coefficients for all predictors, including null findings, in the main text and accompanying table. In addition, we conducted a Bayesian logistic regression with the same model specification as a sensitivity analysis. The Bayesian model showed satisfactory convergence and posterior predictive performance, and the results were consistent with the frequentist analysis. Together, these analyses provide a more complete assessment of the evidence while acknowledging the exploratory nature of the study. 9) *We would recommend moving the demographics table from the Supplementary Material to the Main Text, as this information is very helpful for interpreting the wider results.* We agree that key demographic information is important for interpreting the findings. Accordingly, we have moved a revised summary demographics table containing the principal participant characteristics to the main text. The more detailed demographic information collected as part of the survey has been retained in the Supplementary Material to avoid interrupting the flow of the manuscript and because several variables were not included in the revised analyses. 10) *The clarity of all figures could be improved. Specifically, we recommend using a more distinct colour palette in Figure 1 and including a y-axis label and figure note for Figures 2 and 3 highlighting that this is percentage per group, to avoid incorrect or misleading interpretations.* Thank you for the helpful suggestion. We have reformatted all figures for better contrast and clarity. **Discussion** 11) *The Discussion needs to be substantially re-written to more accurately reflect the conclusions and interpretations that are permissible from the current results:*

*•    The researchers do not ask any questions about the lived experiences of the participants or their perceptions of inclusivity and support. As such, numerous statements throughout the Discussion need to be rephrased and diluted as they cannot be interpreted from the current results.*

*•    Non-disclosure does not inherently mean that participants are stigmatised. Participants may not have felt the need to disclose their diagnoses because they are already well-accommodated. Whilst we agree stigma is an important issue to consider, stigma was not explicitly measured and thus these assumptions cannot be drawn.*

*•    Similarly, you say wider initiatives within the AIMS-2-TRIALS help to reduce stigma, however this was not assessed and thus cannot be known.*

*•    When you discuss wider neurodevelopmental and mental health conditions, it needs to be made clear that these are only examples, as specific conditions were not accounted for in your methodology.*

*•    The qualitative questions specifically focus on participants’ research trajectories and cannot be used to infer their personal sense of satisfaction and belonging.*

*•    Please could the researchers comment on the potential impact of age on the higher prevalence of neurodivergent ECRs, rather than solely focussing on the (untested) link between neurodivergence and lack of career progression support?* We thank the reviewers for these important observations. We have substantially revised the Discussion to better reflect the exploratory nature of the study and to ensure that interpretations remain proportionate to the data. Specifically:

 •    We have removed or substantially rephrased statements that inferred participants' lived experiences, perceptions of inclusivity, or workplace support, as these were not directly assessed by the survey.

 •    We no longer interpret low rates of disclosure as evidence of stigma. Instead, we acknowledge that disclosure decisions may reflect a range of factors, including privacy preferences, workplace culture, and concerns about disclosure, and we identify these as important areas for future research.

 •    We have removed statements suggesting that AIMS-2-TRIALS initiatives reduced stigma, as this was not evaluated in the present study.

 •    We have clarified throughout the Discussion that references to specific neurodevelopmental and mental health conditions are illustrative examples rather than conditions that were individually assessed within the survey.

 •    We have revised the discussion of the open-ended responses to reflect their intended purpose. Rather than inferring participants' sense of satisfaction or belonging, we now describe these responses as providing preliminary insights into research priorities and identify them as a potential avenue for future qualitative investigation.

 •    We have substantially revised the discussion of career stage. In line with the revised analyses, we no longer interpret the findings as evidence of delayed career progression. Instead, we acknowledge that age and cohort effects may contribute to the observed distribution and emphasise that longitudinal research will be required to distinguish between these possibilities. **General**

 12) *In light of the above, it may be apt for the authors to re-phrase their title, to more accurately reflect the current research*. Thank you for this observation. We have changed the title to “Neurodivergent Researchers in Autism Research: A Survey within the AIMS-2-TRIALS Consortium” to accurately describe the study. 13) *It should be noted as a potential conflict of interest that the authors are funded by the AIMS-2-TRIALS they are currently investigating.* Thank you for raising this point. Several authors have been members of, and have received funding through, the AIMS-2-TRIALS consortium. However, the present study was not commissioned by the consortium, and no authors received specific funding to conduct or publish this work. We agree that the authors' involvement within the consortium is relevant context and have clarified this relationship in ‘Declarations’. 14) *We encourage the authors to be consistent in their use of neurodiverse language, in line with current recommendations (e.g., Bottema-Beutel et al., 2021).* Thank you for this suggestions, we have revised the paper based on the attached recommendations.
